# One-time fertilization in flue-cured tobacco: nutrient dynamics, chemical composition and economic performance across different soil textures

**DOI:** 10.3389/fpls.2025.1649093

**Published:** 2025-10-06

**Authors:** Zhen Zhu, Jianwei Peng, Ajuan Zhao, Boran Wu, Chunxia Ding, Bo Li, Yongliang Han

**Affiliations:** ^1^ National Engineering Research Center for Efficient Utilization of Soil and Fertilizer Resources, College of Resource, Hunan Agricultural University, Changsha, China; ^2^ Tobacco Technology Center, Changsha Tobacco Company of Hunan Province, Changsha, China; ^3^ College of Chemistry and Materials Science, Hunan Agricultural University, Changsha, China; ^4^ Funing County Branch Office, Wenshan Tobacco Company of Yunnan Province, Wenshan, China

**Keywords:** tobacco (*Nicotiana tabacum L*.), one-time fertilization, nutrient uptake, fertilizer utilization, economic benefit

## Abstract

**Introduction:**

One-time fertilization is a promising strategy to reduce labor costs and improve efficiency in agriculture. While its benefits are documented in staple crops (e.g., wheat, rice, maize), the efficacy of this approach in high-value crops like flue-cured tobacco and its interaction with soil texture remain poorly understood. Therefore, this study aimed to 1) evaluate the feasibility of one-time basal application of a specialized fertilizer in flue-cured tobacco, and 2) determine how soil texture (loamy vs. sandy) mediates its agronomic and economic efficacy.

**Methods:**

Four treatments were compared: a no-fertilizer control (CK), conventional split fertilization (CF), one-time application of specialized fertilizer (T1), and specialized fertilizer plus a seedling-raising fertilizer (T2).

**Results:**

Results showed that in loamy soil, T1 and T2 significantly enhanced late-stage nitrogen (N) and potassium (K) accumulation, increasing N use efficiency by 54.5~56.7% compared to CF. The economic gains were highly soil-specific. Although both T1 and T2 reduced labor costs, T2 in sandy soil generated the highest net income. It significantly increased production value by 14.8% and the proportion of high-grade tobacco by 16.7%, respectively (p<0.05), compared to CF. This gain was driven by improved leaf quality rather than increased biomass yield.

**Discussion:**

Loamy soil excelled in nutrient retention and utilization efficiency. This study demonstrates that soil texture mediates the success of one-time fertilization. The T2 strategy offers a profitable, labor-saving alternative, especially in sandy soils, providing a scientific basis for soil-specific fertilization policies to optimize productivity and economic sustainability.

## Graphical Abstract

Illustration comparing three fertilizer application methods (CF, T1, T2) on plant growth in loamy and sandy soils. T1 and T2 show improved nitrogen use efficiency (NUE) and net income with T2 having the highest gains. Graphs depicted that fertilizer use efficiency is higher in loamy soils and economic benefit is higher in sandy soils. The economic benefit components were also analyzed to present the cost and net benefit contributions.

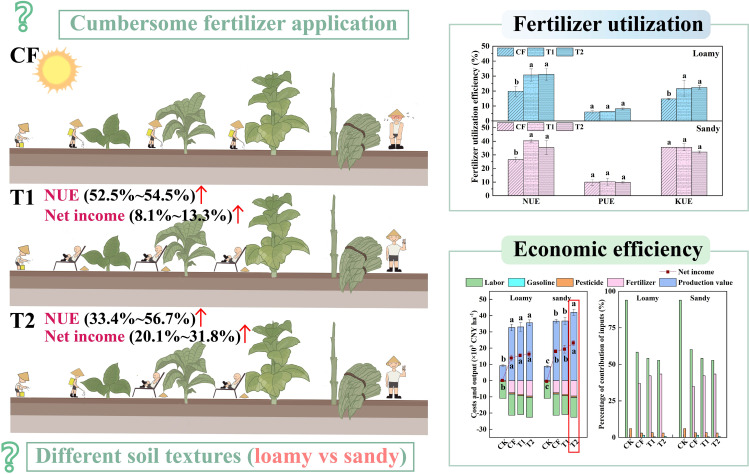

## 1 Introduction

Tobacco (*Nicotiana tabacum L.*) is a globally recognized economic crop. It plays a vital role in boosting national revenue, promoting local economic development, and enhancing the tobacco farmers’ income (Sun et al., 2024). Its yield and quality are predominantly influenced by genetics, fertilization, and cropping systems (Zou et al., 2015; Jiang et al., 2022). Among these, fertilization is paramount, accounting for approximately 39% of yield and 47% of production value (He et al., 2013).

Conventional tobacco cultivation typically involves a base application followed by three to four manual top-dressings to meet crop nutrient demands. However, this practice is increasingly unsustainable. Rising labor costs, driven by a growing shortage of agricultural workers, have significantly increased production expenses (Liu et al., 2018). Furthermore, the pursuit of high yields has led to widespread over-fertilization, which fails to increase output and instead drastically reduces fertilizer use efficiency (Soares et al., 2020; Kumar et al., 2015; Liu et al., 2020; Huang et al., 2023). While strategies like soil-test-based formulation, deep placement, and split applications can mitigate nitrogen losses, they often require additional labor and machinery, further increasing complexity and cost (Hu et al., 2023; Wu et al., 2024, 2023). Therefore, developing a simplified, efficient, and effective fertilization strategy is crucial for the tobacco industry’s sustainability.

One-time fertilization technology, particularly using slow- or controlled-release fertilizers, has emerged as a promising solution to address these challenges. It offers significant potential for labor savings and increased efficiency, demonstrating notable success in staple crops (Zhong et al., 2022; Guo et al., 2017). In wheat, one-time application of controlled-release nitrogen fertilizer has been shown to increase economic returns by 282.4-327.2 yuan ha^-1^ compared to conventional fertilization method (Cui et al., 2022). Similarly, studies in maize (Feng et al., 2021), wheat (Yaseen et al., 2021), and rice (Gil-Ortiz et al., 2020) have reported that one-time fertilization can enhance yield, improve fertilizer use efficiency, and reduce greenhouse gas emissions simultaneously.

Despite these promising results in staple crops, its application in high-value commercial crops like flue-cured tobacco remains underexplored. More critically, even when such strategies are considered, a crucial knowledge gap persists: how soil texture (a fundamental property varying widely across tobacco-growing regions) mediates the efficacy of one-time fertilization remains virtually unknown, given that the efficacy of any fertilization strategy is profoundly influenced by soil properties, particularly texture, which governs water and nutrient retention (Irfan et al., 2018). This interaction is particularly relevant for tailored formulations, such as the specialized blended fertilizer (comprising quick-release and polymer-coated controlled-release nutrients, with a release profile tailored to the growth cycle of flue-cured tobacco) evaluated in this study. Therefore, the novelty of this study lies in its comprehensive evaluation of how soil texture (loamy vs. sandy) mediates the effect of one-time basal fertilization on tobacco nutrient partitioning, utilization efficiency, and most importantly, the economic returns, a critical factor for farmer adoption that has been overlooked in previous studies.

Therefore, we hypothesized that the one-time basal application of specialized fertilizer would optimize nutrient uptake and improve economic returns in flue-cured tobacco, but that these effects would be significantly mediated by soil texture. This study aimed to assess the feasibility of a one-time basal application of specialized fertilizer in flue-cured tobacco by evaluating its effects on nutrient uptake, utilization efficiency, and economic benefits, and determining how these responses are mediated by soil texture (loamy vs. sandy). The ultimate goal is to develop a simplified fertilization strategy that supports sustainable tobacco production.

## 2 Materials and methods

### 2.1 Experimental sites and cultivars

**Figure 1:**
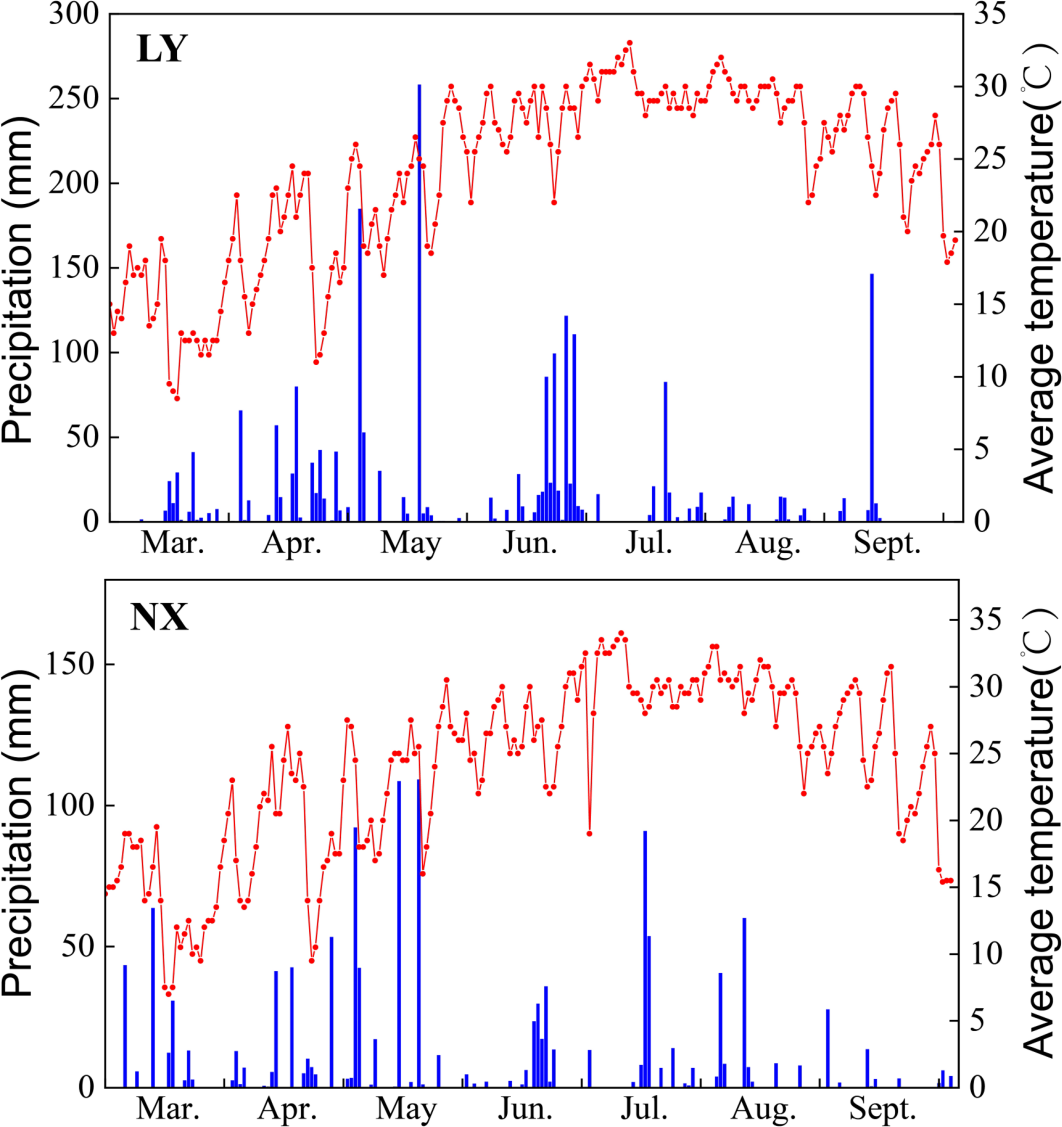
Meteorological data for two regions, LY and NX, during the 2023 tobacco growing season. The left side of the figure shows the average daily rainfall; the right side shows the average daily temperature.

The field experiments were conducted during the 2023 tobacco growing season in Liuyang County (LY) (113°36’ E, 28°29’ N) and Ningxiang County (NX) (112°11’ E, 28°00’ N) in Changsha, Hunan Province, China. Both test areas feature a subtropical monsoon climate. They have four distinct seasons, with plentiful rainfall and high temperatures, which is typical of a humid region. The average daily rainfall and temperature in Liuyang and Ningxiang experimental sites in 2023 are as shown in the **Figure 1**. The soil texture of the LY test field was loamy, while that of the NX test field was sandy. In order to minimize the original spatial variation of nutrients within the fields, the 0–20 cm soil of both test plots was tilled prior to carrying out the experiments, and the land was leveled afterwards. The physical and chemical properties of the original topsoil before the experiment are shown in **Table 1**.

**Table 1:** Primary properties of topsoil (0–20 cm) in Liuyang (LY) and Ningxiang (NX).

Site	Organic matter	TN	TP	TK	alkali-hydrolyzed N	AP	AK	pH	Sand (2-0.05 mm)	Silt (0.05-0.002 mm)	Clay (<0.002 mm)
	g kg-1	g kg-1	g kg-1	g kg-1	mg kg-1	mg kg-1	mg kg-1		%	%	%
LY	17.1	1.3	0.73	23.1	25.3	16.2	79	5.83	34	47	19
NX	18.7	1.5	0.84	26.3	29.5	15.5	87	5.81	86	8	6

In the field experiment, the main tobacco cultivar in China, “Yunyan 87”, was used. Seedings were raised in plastic greenhouses for 40~50 days before being transplanted to the field, and the growing period in the field was 110~115 days. In the LY trial area, tobacco was transplanted on March 18, 2023 and harvested on June 18, 2023. Similarity, in the NX trial area, tobacco was transplanted on March 21, 2023 and harvested on June 19, 2023. After fertilizer application, plastic film was laid along the ridges, and other field management measures were carried out in line with local agricultural practices.

### 2.2 Experimental design

Four treatments were established in this experiment: CK (plots with no fertilizer application, set up for calculating fertilizer utilization), CF (split application of conventional fertilizers, including basal fertilizer, seedling raising fertilizer, and two additional fertilizers), T1 (one-time application of specialized fertilizer), and T2 (specialized fertilizer plus a seedling raising fertilizer). For the three fertilizer-applied treatments (excluding the control), the fertilization levels were kept consistent: 150 kg ha^-1^ of N, 142.5 kg ha^-1^ of P_2_O_5_, 375 kg ha^-1^ of K_2_O. The experiment employed a completely randomized block design with three replications per treatment. Each plot measured 6 m × 12 m (72 m²). Tobacco was planted with a row spacing of 1.2 m and a plant spacing of 0.5 m to ensure uniform growing conditions. A schematic diagram of the block and plot arrangement is provided in **Supplementary Figure S1**.

The fertilizers used in each treatment are provided in **Table 2**, and additional details about the test fertilizers used in the experiment are given in Text S1. The proportion of slow-release fertilizers in tobacco special fertilizers is 30%. These slow-release fertilizers are polyurethane-coated. The slow-release nitrogen fertilizers had a 60-day release period, and the slow-release potash fertilizers had an 80-day release period. The tobacco-specific fertilizer used in this study was provided by the Institute of Agricultural Resources and Regional Planning, Chinese Academy of Agricultural Sciences; while the fertilizer for the conventional fertilization treatment was provided by the Changsha Tobacco Company.

**Table 2:** Description of the details regarding experimental fertilizer application, including the application method and the quantity of fertilizer used.

Treatment	Base fertilizer/(kg ha-1)	Seedling raising fertilizer/(kg ha-1)	Additional fertilizer I/(kg ha-1)	Additional fertilizer II/(kg ha-1)
CK	–	–	–	–
CF	base fertilizer (8.5-10-11) 1231.5	compost fertilizer (20-9-0) 75	KNO3 75	KNO3 150
	calcium-magnesium phosphorus fertilizer 105			
	K2SO4 265.5			
	organic fertilizer 450			
T1	tobacco special fertilizer I (7.8-7.38-18.74) 1923.3	–	–	–
	organic fertilizer 450	–	–	
T2	tobacco special fertilizer II (6.67-6.96-18.05) 2023.8	compost fertilizer (20-9-0) 75	–	–
	organic fertilizer 450			

All the fertilizers used in the experimental treatments of this study are shown in the table above. The organic fertilizers used in the CF, T1 and T2 treatments were all one-time basal applications.

### 2.3 Collection of plant and soil samples

When tobacco grew to the rosette stage (LY: May 4, 2023; NX: May 9, 2023), topping stage (LY: May 26, 2023; NX: May 29, 2023), and harvesting stage (LY: June 18, 2023; NX: June 19, 2023), five plant samples with consistent growth conditions were randomly collected from each experimental plot. These plants were heated at 105°C for 30 minutes to inactivate metabolic processes, and then dried at 80°C until a constant weight was reached. The dry matter accumulation of each part was calculated, and the total N, P and K contents of each part were determined.

At the same time as collecting plant samples, soil samples of 0–20 cm were collected from ten randomly selected points in each plot. Weeds and stones were removed, and the samples were air-dried and through 0.9 mm and 0.154 mm sieves for subsequent analysis of soil physical and chemical properties, including NH_4_^+^-N, NO_3_^–^N, available phosphorus (AP) and available potassium (AK).

The ripening period of tobacco commences from the topping stage. When the lower leaves reach maturity, harvesting is carried out in stages. Depending on the degree of maturity, the tobacco is harvested four times. The harvesting and baking process were conducted following the previous method (Cakir and Cebi, 2010). A CR-300 chromameter (Minolta, Osaka, Japan) was used to assess the maturity of tobacco leaves. When measuring, the main leaf veins were avoided, and five positions on each of the left and right sides of the leaf were selected to measure the leaf color. The measurement index was compared with a standard, and when it exceeded the standard, the tobacco was considered mature (Liu et al., 2013; Kaushik, 2019). After the tobacco leaves matured, they were manually picked from bottom to top in each plot according to their positions and then hung for baking in the corresponding treatments. Representative B_2_F and C_3_F grades of the upper and middle tobacco leaves from each treatment were selected, and 1kg of each was prepared as a composite sample to determine its chemical composition.

### 2.4 Measurement methods

#### 2.4.1 Plant biomass and nutrient content

Plant samples were digested with H_2_SO_4_-H_2_O_2_, the N content in plants was determined using the micro Kjeldahl method (Douglas et al., 1980), the P content was measured by the molybdenum-antimony colorimetric method (Bao, 2000), and the K content was detected via the flame photometric method (Zhu et al., 2013). To ensure the reliability and quality of the data, we used a standard reference tobacco sample (GBW08515) to measure with the collected samples, and three technical replicates were set up for each sample. The spiked standardized recoveries for each element ranged from 90% to 110%. After the tobacco harvest, the fertilizer use efficiency for each treatment was calculated.


$$\begin{array}{l} \text{Accumulation of N},\text{ P and K }\left({\text{kg ha}}^{-1}\right)\\ \;=\text{dry matter weight per plant }\left(\text{g}\right)\times \text{N},\text{ P and K content per plant }\left(\%\right)\\ \;\times \text{ planting density }\left({\text{plant ha}}^{-1}\right)÷1000 \end{array}$$

$$\begin{array}{l} \text{N},\text{ P and K feitilizer utilization }\left(\%\right)\\ =(\text{N},\text{ P and K uptake by aboveground plants in the N},\text{ P and K applied plots}\\ -\text{N},\text{ P and K uptake by abovegrond plants in the control plots)}\\ ÷\text{N},\text{ P and K application }\times 100\% \end{array}$$

#### 2.4.2 Chemical composition of cured tobacco leaves

As described by Hu et al., 2018, the total sugar, reducing sugar, nicotine, total nitrogen, and chlorine contents in the post-roasted tobacco were determined using a continuous flow analyzer (SEAL, Norderstedt, Germany), and the K content in the tobacco was measured by flame atomic absorption spectroscopy (Varian AA-220FS, Thermo Fisher Scientific, USA). Reagents and chemicals were either chromatographic or analytical grade. Millipore ultrapure water (Type I) was used for all analyses. Based on previous studies, a table of suitable ranges of chemical composition of high-quality tobacco (**Table 3**) was established to evaluate the post-roasted tobacco of each treatment (Yan, 2002; Hu et al., 2021; Wang et al., 2022). Values within the appropriate range were assigned a score of 1, while those outside the range were assigned a score of 0. The final score of the chemical composition quality of the sample was obtained by averaging the scores of all indicators in the sample and then multiplying by 100.

**Table 3:** Appropriate range of chemical composition of high-quality tobacco.

Indicator	Range (upper leaf)	Range (middle leaf)
Total sugar (%)	20~25	23~29
Reducing sugar (%)	16~21	20~23
Nicotine (%)	3~3.5	2.0~3.0
K (%)	>2	>2
Chlorine (%)	0.3~0.6	0.3~0.6
Total nitrogen (%)	1.6~2.8	1.4~2.2
Sugar-alkali ratio	6~10	8.5~13.5
Nitrogen-alkali ratio	0.6~0.8	0.7~1.0
K-chlorine ratio	≥4	≥4

#### 2.4.3 Soil

Soil mineral nitrogen (in the forms of NH_4_^+^ and NO_3_^-^) was extracted using 0.01 M CaCl_2_ solution and analyzed with a continuous-flow analyzer (SmartChem450, AMS Alliance) (Hei et al., 2023). Soil AP was extracted with 0.5 M NaHCO_3_ solution, while soil AK was extracted with 1 M NH_4_OAc solution. The AP content was determined by molybdate blue colorimetry, and the AK content was determined by flame photometry, as per the procedures reported by Hei et al. (2022). Standard soil samples (GBW07387, GBW07458) from the National Center for National Standard Substances of China were used as quality control during the analysis. Three technical replicates were set up for each sample analysis.

#### 2.4.4 Economic analysis

The roasted tobacco was sorted according to the hanging mark. Subsequently, the quality inspectors of the local tobacco company graded and weighed the tobacco of each treatment in accordance with the GB/T2635 tobacco grading standard. Then, the yield (biological yield of ungraded tobacco), production value, average price, and proportion of top-grade cigarettes of each treatment were calculated.


$$\begin{array}{l} \text{Production value }\left({\text{yuan ha}}^{-1}\right)\\ \;=\text{weight of each grade of tobacco }\left({\text{kg ha}}^{-1}\right)\\ \;\times \text{ unit price of correspongding grade of tobacco }\left({\text{yuan ha}}^{-1}\right) \end{array}$$

$$\text{Average price}({\text{yuan kg}}^{-1})=\text{production value}({\text{yuan ha}}^{-1})÷\text{production}({\text{kg ha}}^{-1})$$

$$\begin{array}{l} \text{Proportion of top grade tobacco }\left(\%\right)\\ =\text{weight of top grade tobacco }\left(\text{kg}\right)÷\text{total prodution }\left(\text{kg}\right)\times 100\% \end{array}$$

$$\begin{array}{l} \text{Net in come }(\text{yuan }{\text{ha}}^{\left(-1\right)})\\ =\text{value of tobacco production }(\text{yuan }{\text{ha}}^{\left(-1\right)})\\ -\text{cost of production }(\text{labor cost and materialized cost})(\text{yuan }{\text{ha}}^{\left(-1\right)}) \end{array}$$

Here, the production value calculations were based on graded, marketable tobacco. The unit prices for different grades were derived from the official fixed purchase price system established by the local tobacco company for the 2023 season. The specific unit prices are shown in **Supplementary Table S1**. Labor costs were estimated based on the prevailing local daily wage rate of 200 yuan per person-day for agricultural work in the 2023 season. Labor costs mainly comprised the expenses associated with various field management tasks such as film-covering, film-uncovering, soil cultivating, hoeing, topping, removing bottom leaves, fertilization, transplanting, pest control, and post-harvest operations like leaf picking, baking and grading. Material and chemical costs mainly include the expenditures for purchasing seedlings, fertilizers, pesticides, fuel and other input costs. The sum of labor cost and materialized cost constituted the total production cost of tobacco cultivation.

### 2.5 Statistical analysis

Data were organized using Microsoft Excel 2016. All statistical analysis were performed using IBM SPSS Statistics (Version 22.0). Treatment means were compared using a one-way analysis of variance (ANOVA). When the ANOVA indicated a significant effect (p<0.05), *post-hoc* comparisons were performed using the Least Significant Difference (LSD) test. Subsequently, the data were plotted using Origin 2019 pro. The data presented in tables and figures are expressed as the mean ± standard deviation (SD) of all replicates. Correlation analysis and random forest analysis were performed using the “psych” and “random forest” R packages to explore the relationships among nitrogen uptake, yield, nitrogen fertilizer utilization, chemical coordination, and economic efficiency of tobacco plants.

## 3 Results

### 3.1 Distribution of N, P and K accumulation in different parts

**Figure 2:**
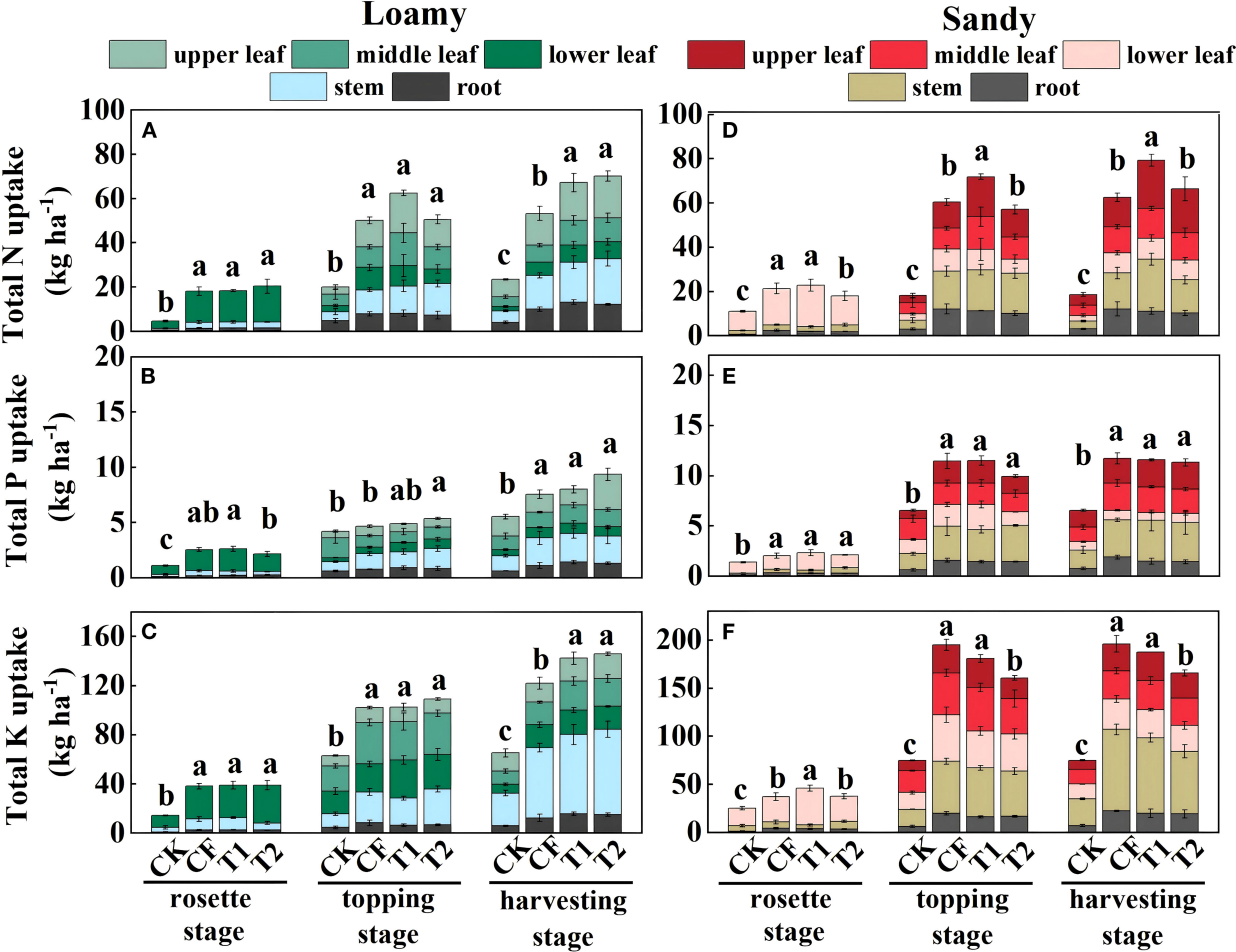
N **(A, D)**, P **(B, E)**, and K **(C, F)** accumulation in different parts of the tobacco plant. Data are presented as means ± SE (n=3). The value of n represents the number of experimental replicates analyzed for each treatment group. Different lowercase letters above the error bars in the graphs represent significant differences between treatments by ANOVA test (p<0.05).

The results indicated that in loamy soil, the accumulation of N, P and K nutrients in tobacco plants increased as the tobacco grew (**Figure 2**). At the rosette and topping stages, there were no significant differences in N, P and K accumulation among the fertilizer treatments. However, at the harvesting stage, compared with total N accumulation (53.26 kg ha^-1^) and total K accumulation (122.03 kg ha^-1^) in CF, T1 and T2 increased by 26.18%~31.74% (p=0.03, p=0.01) and 16.65%~19.55% (p=0.04, p=0.02), respectively, and the differences between T1 and T2 were not significant. T2 treatment exhibited the highest total N and total K accumulation in roots, stems, middle leaves and upper leaves, which was significantly higher than that of CF (**Figures 2A, C**).

In contrast to loamy soil, in sandy soil the increase in total N and K accumulation in tobacco plants slowed down after the rosette stage (**Figures 2D, F**), while P accumulation remained relatively stable. At the topping (p=0.04) and harvesting stages (p=0.01) (**Figure 2D**), the total N accumulation in T1 was significantly higher than that of CF.

### 3.2 Distribution ratio of N, P and K accumulation at different growth stages

**Figure 3:**
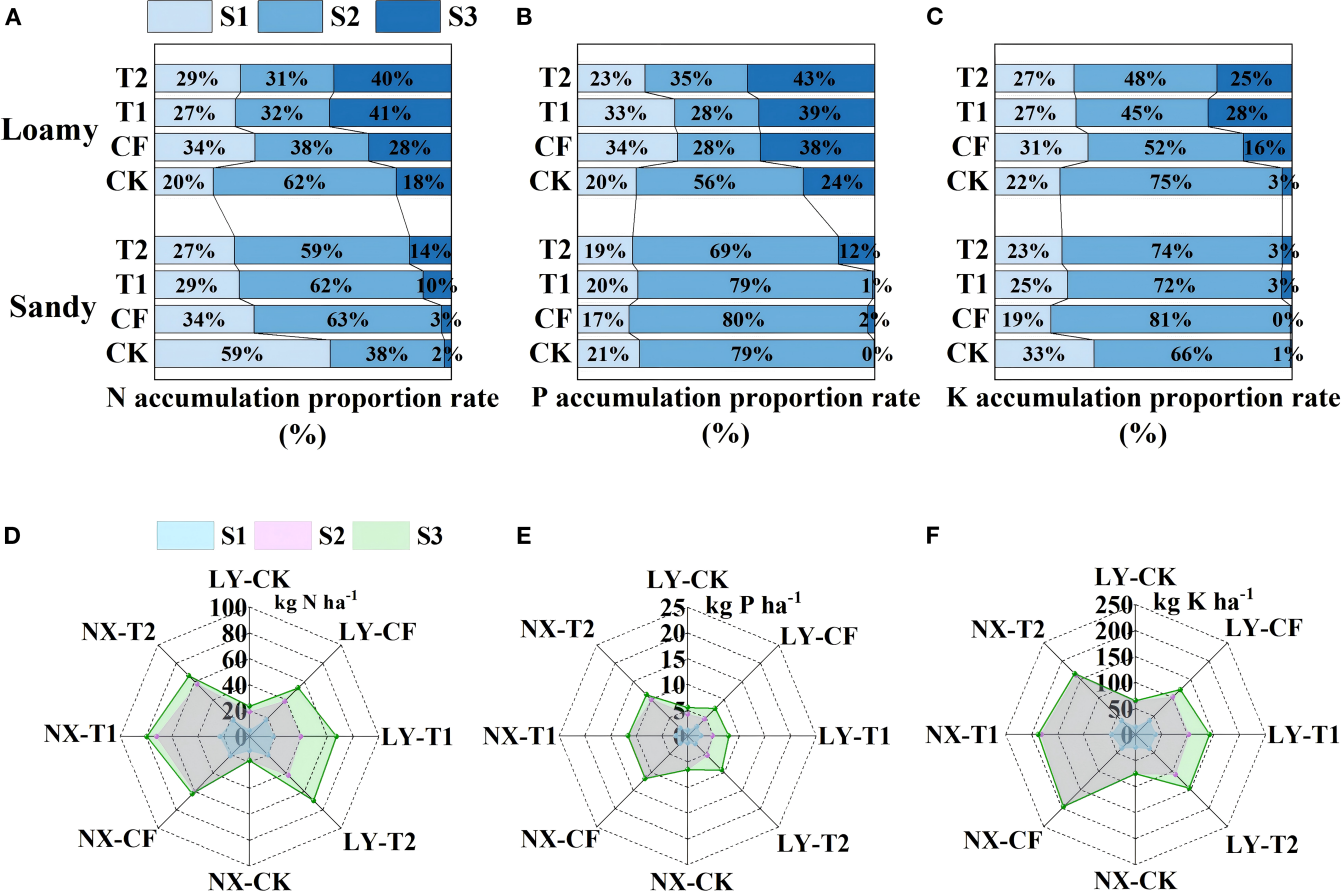
Percentage and accumulation of N **(A, D)**, P **(B, E)** and K **(C, F)** at different growth stages (S1、S2、S3) of the tobacco plant. S1: transplanting-rosette stage, S2: rosette-topping stage, and S3: topping -harvesting stage. Data are presented as means ± SE (n=3). The value of n represents the number of experimental replicates analyzed for each treatment group.

As depicted in **Figure 3A**, in loamy soil, tobacco plants from T1 and T2 treatments still accumulated up to 40% N at the S3 stage (topping-harvesting). Phosphorus was mainly accumulated at the S1 stage (transplanting-rosette) (accounting for 74%~78%) (**Figure 3B**), while K was mainly accumulated (45%~52%) at the S2 stage (rosette-topping) (**Figure 3C**), with less accumulation in the remaining stages. In contrast, in sandy soil, nitrogen in tobacco plants was mainly accumulated during the S2 stage (59%~63%) and less N was accumulated at the S3 stage (topping-harvesting) (**Figure 3A**).

In loamy soil, N accumulation in T1 and T2 at S3 stage was 27.57 kg ha^-1^ and 27.73 kg ha^-1^ respectively, which was 86.29% and 87.37% higher than that of conventional fertilizer application (**Figure 3D**). The K accumulation was 40.01 kg ha^-1^ and 36.73 kg ha^-1^ respectively, 100.44% and 84.04% higher than that of conventional fertilizer application (**Figure 3F**). In sandy soil, compared with CF, T1 and T2 treatments could still increase the N and K accumulation of tobacco plants at the S3 stage. Moreover, in both loamy and sandy soils, tobacco plants absorbed P continuously and steadily throughout the reproductive stage, with more accumulation at the S1 stage, less at the S2 stage, and still some accumulation at the S3 stage (**Figure 3E**).

### 3.3 Effect of different fertilizer application methods on fertilizer utilization rate

**Figure 4:**
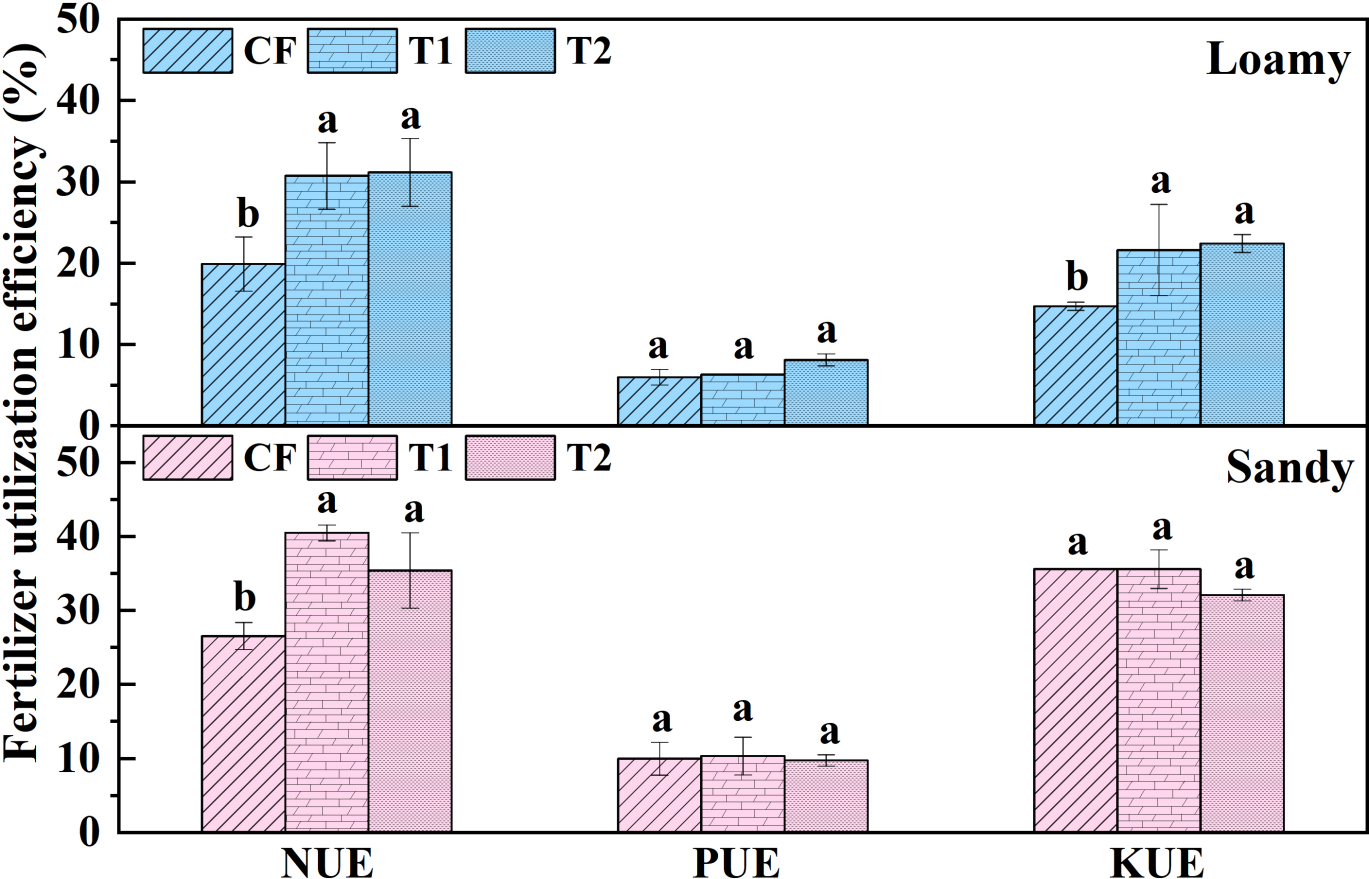
N, P and K fertilizer utilization of different fertilization methods. Data are presented as means ± SE (n=3). The value of n represents the number of experimental replicates analyzed for each treatment group. Different lowercase letters above the error bars represent significant differences between treatments by ANOVA test (p<0.05). NUE, NUE and NUE in the figure represent nitrogen fertilizer use efficiency, phosphorus fertilizer use efficiency and potash fertilizer use efficiency, respectively.

As can be observed from **Figure 4**, compared with CF, T1 and T2 treatments in loamy soil had the highest N-fertilizer utilization efficiency, reaching 30.72% and 31.15% respectively. This was significantly higher than CF by 54.53%~56.69% (p=0.01, p=0.01). The K-fertilizer utilization efficiencies of T1 and T2 treatments in loamy soil were 21.6% and 22.4% respectively, which was significantly higher than CF by 46.94%~52.38% (p=0.0003, p=0.003). In sandy soil, the N-fertilizer use efficiency of T1 and T2 treatments was significantly increased by 33.41%~52.54% (p=0.01, p=0.01), but there was no significant difference in K-fertilizer use efficiency among the treatments. In both soils, there were no significant difference in P-fertilizer utilization efficiency among all fertilizer treatments. These results indicated that one-time application of tobacco-specific fertilizer could significantly improve the N-fertilizer utilization efficiency of flue-cured tobacco, which is conducive to reducing the loss of nitrogen fertilizer.

### 3.4 Dynamics of soil available nutrients at different growth periods

**Figure 5:**
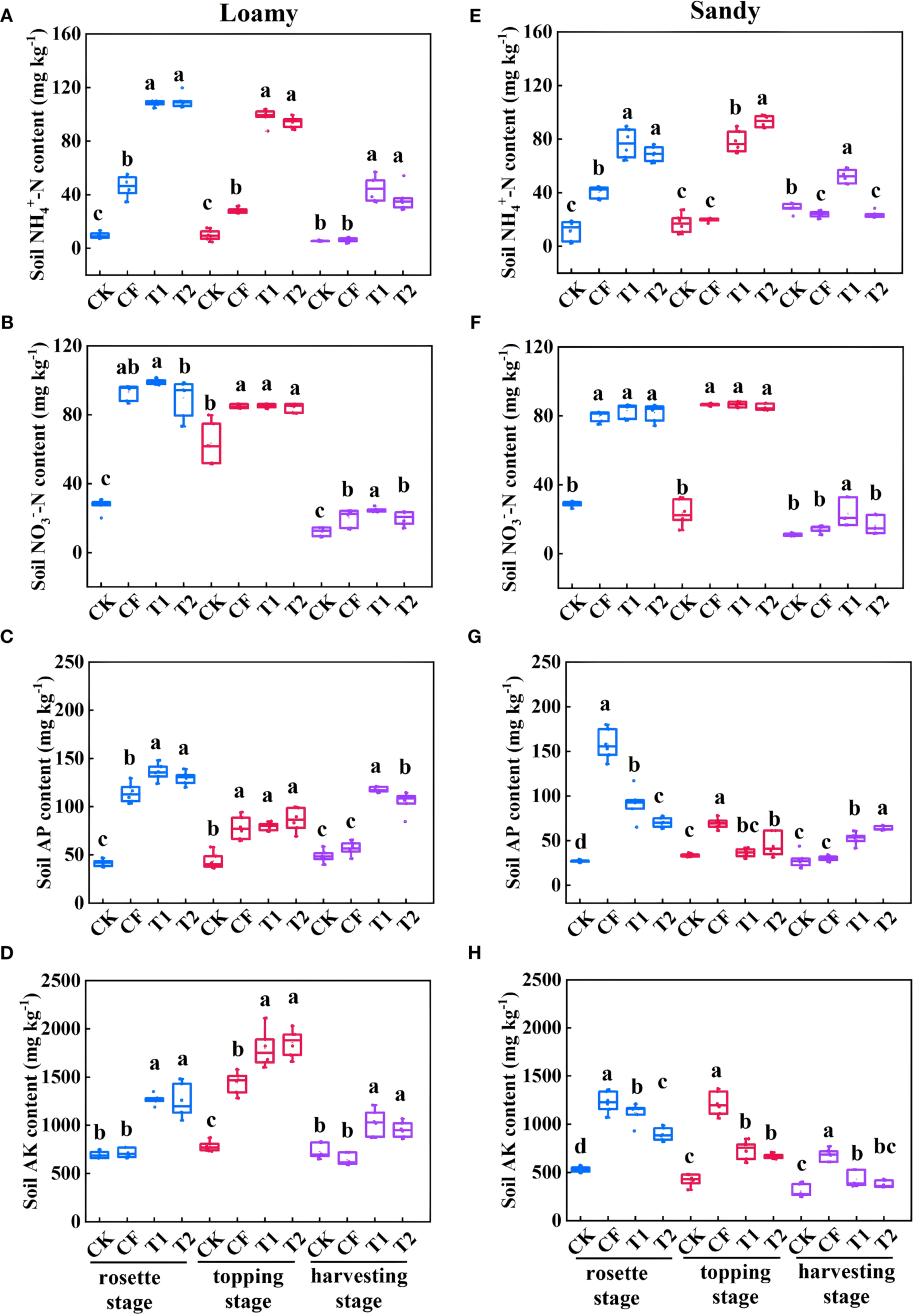
Soil NH_4_^+^-N **(A, E)**, NO_3_^-^-N **(B, F)**, AP **(C, G)**, and AK **(D, H)** contents of treatments at different growth periods. Data are presented as means ± SE (n=3). Lowercase letters above the error bars indicate that the ANOVA test showed a significant difference between treatments (p<0.05).

As shown in **Figure 5**, in loamy soil, the contents of soil NH_4_^+^-N and AK in T1 and T2 treatments were significantly higher than those of CF treatment at different growth stages (p<0.05) (**Figures 5A, D**). The soil NO_3_^–^N content in T1 treatment was significantly higher than that in CF at the harvesting stage (p=0.03) (**Figure 5B**). The soil AP content followed the order: T1 (117.61 mg kg^-1^) > T2 (105.11 mg kg^-1^) > CF (56.77 mg kg^-1^) (**Figure 5C**). In sandy soils, at the rosette and topping stages, the soil NH_4_^+^-N and NO_3_^–^N contents in T1 and T2 treatments were significantly higher than those in CF (p<0.05). At the harvesting stage, the soil NH_4_^+^-N (52.23 mg kg^-1^) and NO_3_^–^N (23.44 mg kg^-1^) were highest in T1 treatment (**Figures 5E, F**). The AK content in sandy soil showed a different trend from that in loamy soil, with CF treatment having the highest AK content, which was significantly higher than T1 (p=0.0001) and T2 (p=0.0001) (**Figure 5H**). At harvesting stage, the soil AP content followed the order: T2 (63.91 mg kg^-1^) > T1 (52.53 mg kg^-1^) > CF (30.18 mg kg^-1^) (**Figure 5G**).

### 3.5 Effect of fertilizer application methods on chemical quality of roasted tobacco leaf

**Figure 6:**
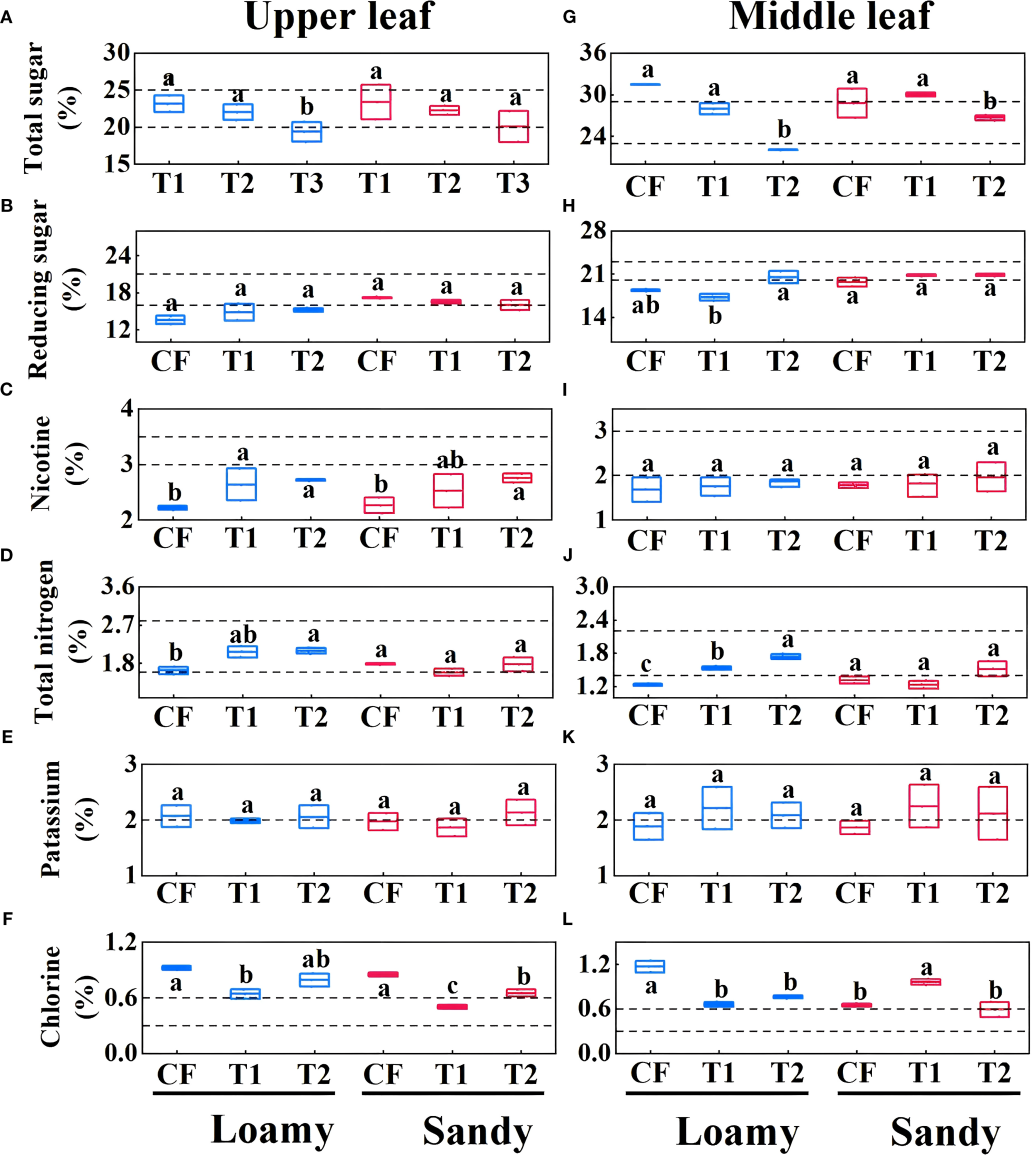
Chemical quality content (total sugar, reducing sugar, total nitrogen, potassium and chloride) of upper leaf **(A–F)** and middle leaf **(G–L)** of different fertilization methods. Data are presented as means ± SE of three replicates. Significant differences (p<0.05) as determined by one-way ANOVA test are indicated by different lowercase letters.

From **Figure 6**, it can be seen that in loamy soil, the total sugar content of the upper and middle leaf in CF treatment was significantly higher than that of the T2 treatment (p=0.008, p=0.001), and the chlorine content was higher than that of T1 treatment in both cases (p=0.03). Conversely, the total N contents of upper and middle leaf and the nicotine contents of the upper leaf in T1 and T2 treatments were significantly higher than the corresponding contents in CF (p<0.05) (**Figures 6C–J**). In sandy soil, the total sugar contents of middle leaf in CF treatment were significantly higher than those in T2 treatment (p=0.03), while they did not differ from those in T1 treatment (p=0.19) (**Figure 6G**). The nicotine contents of upper leaf in T2 treatment were significantly higher than that in CF treatment(p=0.02) (**Figure 6C**). The chlorine contents of the upper leaf in CF treatment and the middle leaf in T1 treatment were the highest among all treatments, but neither of them was within the suitable range (**Figures 6F, L**).

**Figure 7:**
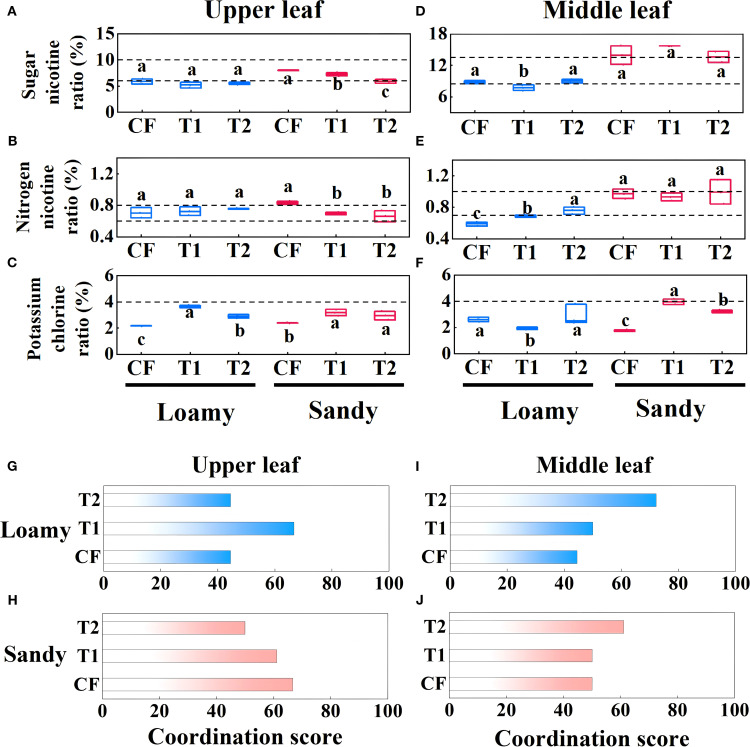
Effects of different fertilizer applications on chemical concordance (sugar-alkali ratio, nitrogen-alkali ratio, and potassium-chloride ratio) of upper leaves **(A–C)** and middle leaves **(D–F)**, and on chemical quality scores **(G–J)** of upper and middle leaves. Data are presented as means ± SE of three replicates. Significant differences (p<0.05) as determined by one-way ANOVA test are indicated by different lowercase letters.

In loamy soil, except for the middle leaf, the potassium-chlorine ratios of the upper and middle leaf in CF treatment were significantly lower than the corresponding ratios in T1 and T2 treatments. The nitrogen-alkali ratios of the middle leaf in CF were also significantly lower than those in T1 and T2 treatments (p<0.05) (**Figures 7A–F**). In loamy soil, T1 treatment had the highest chemical quality score for upper leaf, which was 50.02% higher than the score of CF treatment. For the middle leaf, the trend of chemical quality scores for fertilizer treatments was T2 (72.22%) > T1 (50.00%) > CF (44.44%). In sandy soil, CF treatment had the highest chemical quality score for the upper leaf, while T2 treatment had the highest score for the middle leaf, which was 22.22% higher than the score of CF treatment (**Figures 7G–J**).

### 3.6 Effect of fertilizer application methods on the economic efficiency of baked tobacco production

As presented in **Table 4**, in both loamy and sandy soils, there were no significant difference in the biological yield of tobacco between T1, T2 and CF. In loamy soil, although 8.74% increase in production value was observed in T2 treatment compared to CF treatment, there was no significant difference (p>0.05), which is consistent with the results of proportion of top-grade tobacco and average price. On the contrary, in sandy soil, T2 treatment showed a significant 14.84% (p<0.05) increase in tobacco production value and 16.70% (p<0.05) increase in proportion of top-grade tobacco compared to CF treatment. In addition, production value, proportion of top-grade tobacco and average price were significantly higher by 14.57%, 15.40% and 5.64% in the T2 treatment compared to T1 (p<0.05), and the difference between CF and T1 treatments was not significant. Specifically, our analysis showed that the increase in production value was a result of the improvement in tobacco quality (grade structure change).

**Table 4:** Economic properties of tobacco leaves after roasting with different fertilizer applications.

Soil texture	Treatment	Yield (kg ha^-1^)	Proportion of top-grade tobacco (%)	Average price (yuan kg^-1^)	Production value (yuan ha^-1^)	ΔCF (%)
Loamy	CK	643.39 ± 17.08 b	7.71 ± 0.79 c	17.65 ± 1.04 b	9233.40 ± 468.45 b	–
	CF	1803.10 ± 79.41 a	49.84 ± 1.28 a	29.45 ± 1.21 a	32713.65 ± 2046.79 a	–
	T1	1793.17 ± 56.60 a	48.20 ± 0.51 b	29.99 ± 0.57 a	33078.79 ± 2730.12 a	1.12
	T2	1889.23 ± 66.86 a	49.19 ± 0.46 ab	30.08 ± 0.36 a	35572.80 ± 1786.22 a	8.74
Sandy	CK	684.74 ± 36.85 b	6.67 ± 2.16 c	17.59 ± 1.41 c	8716.67 ± 547.40 c	–
	CF	1922.20 ± 142.10 a	48.54 ± 2.05 b	30.01 ± 0.55 ab	36613.43 ± 1122.02 b	–
	T1	1906.35 ± 179.56 a	49.09 ± 1.75 b	29.72 ± 0.66 b	36701.85 ± 2346.88 b	0.24
	T2	2125.79 ± 37.75 a	56.64 ± 2.39 a	31.39 ± 0.10 a	42048.84 ± 1899.37 a	14.84

Yield means the biological yield of all ungraded tobacco. Amounts in the table for average price and production value are in Chinese Yuan, CNY. Data are presented as means ± SE (n=3). The value of n represents the number of experimental replicates analyzed for each treatment group. The different letters after values in the same column indicate significant difference by ANOVA test (p<0.05). ΔCF (%): The percentage increase in production value for T1 and T2 treatments compared to the CF treatment. It was calculated as: (Production value of Tn - Production value of CF) / Production value of CF × 100%.

**Figure 8:**
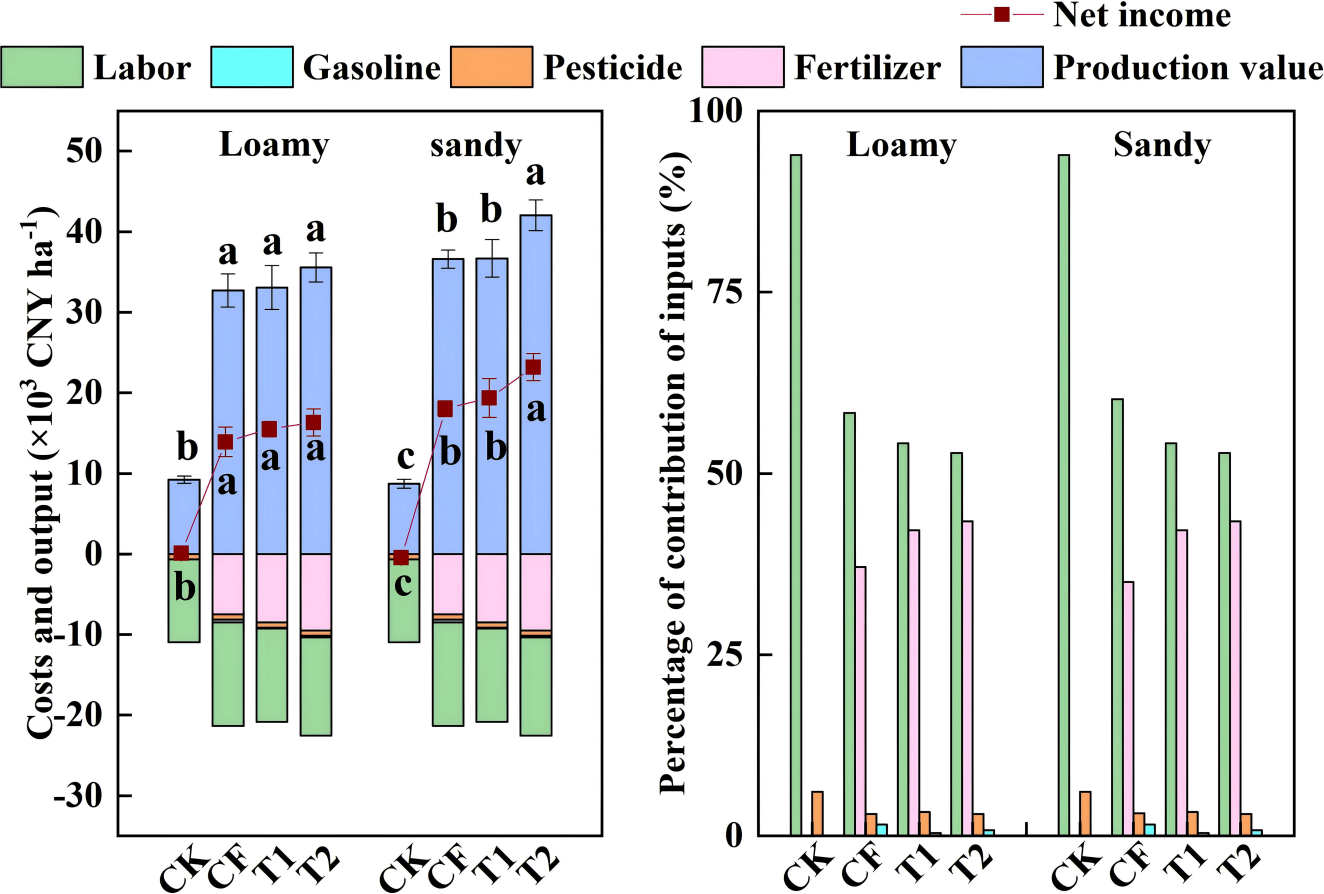
Inputs, production value and net income of different fertilizer application. Data represent means ± SE of three independent replicates. Significant differences (p<0.05, ANOVA) are indicated by different letters.

Furthermore, **Figure 8** indicated that labor cost accounted for the largest proportion of traditional tobacco production costs, representing 60.25% and 58.34% of the total cost in loamy and sandy soils respectively, followed by fertilizer cost, which accounted for 35.03% and 37.09% of the total cost. In loamy soil, the net economic benefits of post-roasting tobacco in T1 and T2 were 12460 CNY ha^-1^ and 13690 yuan ha^-1^ respectively, which were 16.76% (p=0.150) and 28.30% (p=0.036) higher than that in CF. In sandy soil, the net economic benefits of post-roasting tobacco in T1 and T2 were 15860 yuan ha^-1^ and 19490 yuan ha^-1^ respectively, which were 3.86% (p=0.380) and 27.64% (p=0.007) higher than that in CF. Overall, the one-time application of special tobacco fertilizer and special fertilizer plus seedling raising fertilizer can reduce costs, increase efficiency, and enhance the economic benefits in tobacco production.

### 3.7 Reliable predictors of fertilizer utilization and economic efficiency

The N-fertilizer utilization was positively correlated (p<0.05) with the N uptake accumulation in the stems, lower leaves, and upper leaves of tobacco plants, as well as the soil NH_4_^+^-N content. The net economic benefits in tobacco production were positively correlated (p<0.05) with the proportion of production value, top-grade tobacco, average value, input, nitrogen/nicotine ratio in the middle leaf, soil NO_3_^–^N content, N uptake accumulation in all parts of the tobacco plant (roots, stems, lower leaf, middle leaf, and upper leaf), and tobacco yield. Random forest analysis identified soil NH_4_^+^-N (10.09%), stem N accumulation (7.31%), and upper leaf N accumulation (7.06%) as significant predictors (p<0.05) of N-fertilizer utilization. Tobacco production value (12.65%), yield (7.61%), middle nitrogen/nicotine ratio (4.34%), middle sugar/nicotine ratio (4.11%) and soil AK (3.78%) as the highest predictors of net economic efficiency of tobacco production (p<0.05).

## 4 Discussion

### 4.1 One-time fertilization pattern enhances nutrient accumulation and utilization

This study demonstrates that one-time basal application of specialized fertilizers (T1, T2) significantly enhanced nitrogen accumulation in flue-cured tobacco, primarily through increased nutrient partitioning to upper leaves (**Figures 2A, D**). In loamy soil, T1 and T2 treatments significantly improved nitrogen use efficiency (NUE) by 54.53%-56.69% and potassium use efficiency by 46.94%-52.38%, while in sandy soil, significant improvement was observed only for N utilization (p<0.05) (**Figure 4**). These findings are consistent with previous reports in other crops: compared to split applications, one-time application of controlled-release fertilizers increased nitrogen accumulation and NUE in wheat and rice (Cui et al., 2022; Zhao et al., 2021), and promoted nitrogen allocation to vegetative organs (leaves) in cotton (Liu et al., 2022).

**Figure 9:**
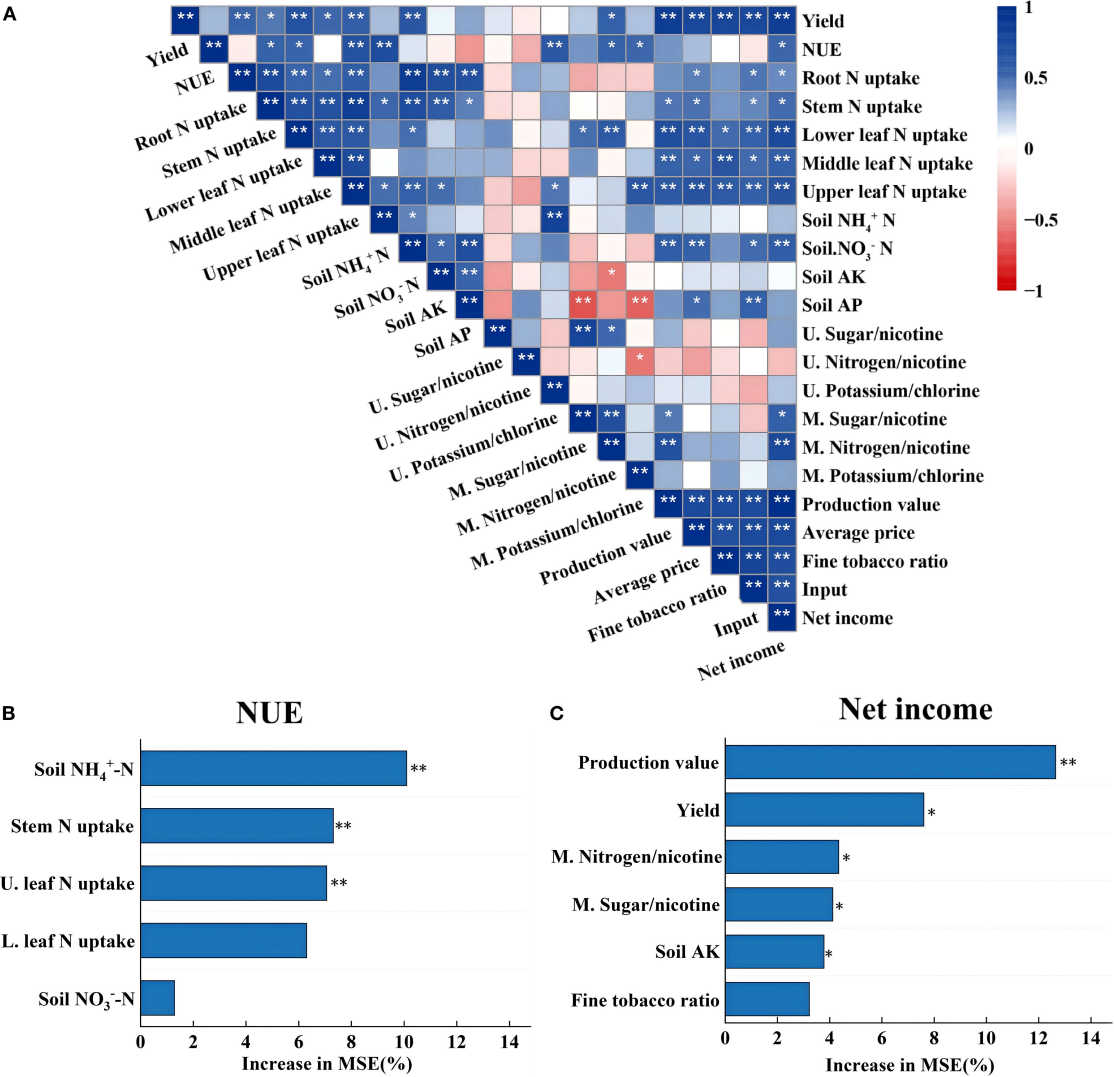
Relationships among tobacco plant N uptake, yield, N fertilizer utilization, chemical coordination, and economic efficiency **(A)**. The main predictors of tobacco N fertilizer utilization **(B)** and net income **(C)** were determined by a random forest model. Asterisks indicate significance at the p<0.05 and p<0.01 significance levels (denoted by * and **, respectively).

The improvement in nutrient use efficiency may be largely attributed to the optimized nutrient release dynamics of controlled-release fertilizers, which better synchronize with crop demand. This mechanism is further corroborated by the elevated soil NH_4_^+^-N levels and enhanced nutrient accumulation in upper leaves and stems under T1 and T2 treatments (**Figure 9**). Controlled-release blended fertilizers improve nutrient effectiveness through steady nutrient supply, reducing the need for frequent applications, while simultaneously regulating rhizosphere soil enzyme activities and promoting nutrient transformation processes (Li et al., 2023; Ahmad et al., 2023; Chamoli et al., 2024; Ahmad et al., 2025; Li et al., 2021), thereby synergistically enhancing plant growth and nutrient uptake. Meanwhile, it have been shown to increase mineral N retention in topsoil, reduce N leaching into deeper layers, and lower N_2_O emissions, thereby significantly improving fertilizer use efficiency and crop yield in wheat systems (Ma et al., 2023, 2021; Thilakarathna et al., 2020). One-time application of specialized controlled-release fertilizers represents an effective strategy for optimizing nutrient supply, improving use efficiency, and supporting sustainable tobacco production.

### 4.2 Soil texture determines the efficiency of nutrient retention and utilization

Different soil textures significantly influence N uptake and utilization by tobacco (Zheng et al., 2021) by governing soil moisture, temperature, and particle-fertilizer contact, which crucially affect nutrient release and retention (Grant et al., 2012; Yang et al., 2016). Specifically, loamy soils with greater cation exchange capacity (CEC) and abundance of micropores enhance NH_4_^+^-N fixation and NO_3_^−^ retention, whereas sandy soils with low water-holding capacity and high hydraulic conductivity predispose them to water loss and nutrient leaching (Cordeiro et al., 2022; Whetton et al., 2022; Meng et al., 2022). Consistent with these principles, our results demonstrated a clear soil-texture-mediated divergence in nutrient fate: in loamy soil, treatments T1 and T2 maintained significantly more NH_4_^+^-N(**Figure 5**), and higher levels of TN and TK at maturity than CF (p<0.05) (**Supplementary Table S2**). They also accrued up to 40% of their total N during the late harvesting stage (**Figure 3A**). Conversely, tobacco plants in sandy soil showed markedly reduced late-stage N accumulation. However, it is important to note that the higher NH_4_^+^-N pool could also indicate a suppression of nitrification processes or an increased risk of NH_3_ volatilization, which were not measured in this study.

Furthermore, potassium (K) dynamics exhibited a distinct challenge in sandy soils. Beyond lower total K accumulation, the K-fertilizer utilization efficiency of T1 was also reduced compared to CF. We speculate that under the typical wet-dry cycles of sandy soils, K^+^ becomes immobilized within clay mineral lattices (Akcura et al., 2019; Bilen et al., 2019), rendering it unavailable for plant uptake and explaining why the one-time application failed to sustain K supply.

### 4.3 Effects of one-time fertilization on tobacco leaf quality and chemical composition

As an economic crop, the quality and chemical composition of tobacco leaves are as crucial as the yield. N not only determines the growth and development of tobacco but also plays a significantly influences tobacco yield and quality, similar to other field crops (Salehzadeh et al., 2016). Tobacco is known to utilize both NH_4_^+^ and NO_3_^-^ or AN for proper growth and development (Fan et al., 2018). While nitrate sources are important for yield, it has been observed that the treatments with the highest tobacco yields tend to have the lowest quality, especially in flavored tobacco, where yield and quality may exhibit an inverse relationship (Kurt and Kinay, 2021). Consistent with these findings, in our study, the CF treatment resulted in the highest biological yield of upper leaves but the lowest chemical quality score after roasting. K is another essential element strongly associated with tobacco quality. It supports the growth of flue-cured tobacco (Subhashini, 2016; Gu et al., 2018) and may contribute to the aroma quality and smoking characteristics by influencing the synthesis of aromatic hydrocarbons compounds (Sakaki et al., 1984, 1985a; Sakaki et al., 1985b). High-quality tobacco typically contains over 2.5% K, and low K levels can adversely affect chemical composition and final product quality (Zhang et al., 2024). In this study, specialized fertilizers showed a potential slow-release effect in loamy soil, which has higher water and nutrient retention capacity. This may better meet the K demand of tobacco during the later growth stages. In contrast, sandy soil, with its higher risk of nutrient leaching, might lead to insufficient K supply toward maturity, possibly affecting sensory and commercial qualities such as taste, aroma, and oil content. K content in tobacco is generally negatively correlated with nicotine and chloride content and positively correlated with total and reducing sugars (Tso et al., 1960). In both soil types examined here, the K, total sugar, and reducing sugar contents of tobacco leaves fell within appropriate range, while the nicotine was below the optimal range. Sugars are involved in synthesizing proteins, nucleic acids, lipids, and aroma compounds, and serve as energy sources during growth and development (Deng et al., 2009). Notably, in sandy soil, the chemical quality score of upper leaves in T2 was lower than that in CF and T1, which may related to the total sugar content exceeding the appropriate range (**Figure 5**). Nevertheless, for middle leaf across both soil types, the T2 treatment resulted in the highest chemical quality scores. These findings suggest that the combined application of specialized fertilizer and seedling-raising fertilizer could be more conducive to producing tobacco leaves with improved industrial quality.

### 4.4 Economic benefits of one-time fertilization are dictated by soil texture

Economic evaluation is critical from the perspective of farmer profitability. Although controlled-release fertilizers (CRF) entail higher initial material costs, these can be partially offset by reduced labor requirements associated with top-dressing (Liu et al., 2014; Guo et al., 2017). Previous studies have shown that compared to conventional cultivation, CRF treatments saved 118.8 CNY ha^-1^ of total inputs, and CRF-60 or CRF-80 treatments achieved higher economic returns due to higher yields (Cui et al., 2022). Consistent with these findings, our study showed that although the T2 treatment increased total input cost by 572.4 yuan ha^−1^, it raised production value by 2859.2 yuan ha^−1^ in loamy soil and 5435.4 yuan ha^−1^ in sandy soil, resulting in the highest economic efficiency among all treatments (p<0.05) (**Figure 8**). By contrast, although T1 reduced both labor and total input cost, it failed to significantly improve production value. A random forest analysis further confirmed that production value was the primary determinant of net return (**Figure 9**). These results collectively suggest that a one-time basal application of specialized fertilizer combined with seedling-raising fertilizer offers a profitable fertilization strategy for tobacco production.

Notably, the improved economic outcome under T2 was not driven by increased biological yield, which did not differ significantly among treatments, but primarily by enhanced leaf quality. This quality-driven effect was particularly pronounced in sandy soil. The mechanism underlying this quality improvement in sandy soil is likely tied to its inherent nutrient dynamics. The limited nutrient retention capacity of sandy soil, often a agronomic challenge, likely promoted a more timely nitrogen consumption in the late growth stage. This process facilitates the critical shift from nitrogen to carbon metabolism, enhancing the accumulation of sugars and aroma precursors essential for superior curing quality and market-grade leaf development (Schlüter et al., 2012; Zhang et al., 2018). Consequently, under sandy soil conditions only, T2 significantly increased the proportion of top-grade leaves, thereby raising the average sales price per kilogram, leading to a significant increase in economic output value. By contrast, in loamy soil, the specialized fertilizer maintained yield and quality without compromising economic returns, demonstrating its broader adaptability as a labor-saving option.

In conclusion, our findings highlight that the economic benefit of one-time fertilization is not universal but is critically dependent on soil texture. This dictates a precision agronomic strategy: prioritizing specialized fertilizers in sandy soils to maximize economic return through quality premium, while utilizing them in loamy soils primarily for their labor-saving reliability.

### 4.5 Limitations and future perspectives

This study has limitations that warrant consideration. First, the findings are derived from a single growing season and one tobacco cultivar; therefore, leaving inter-annual and genotypic variations in fertilizer response unexplored. Second, while mechanisms are inferred, direct quantification of environmental processes (e.g., N_2_O emissions, NH_3_ volatilization, nutrient leaching) and underlying microbial dynamics (e.g., nitrification inhibition) was beyond our scope. Future research should thus prioritize multi-year trials, diverse genetic materials, and comprehensive environmental and microbial analyses to build a more robust understanding.

Despite these limitations, the central finding-that soil texture mediates the efficacy of one-time fertilization, has implications beyond tobacco cultivation. This strategy could be extended to other high-value, labor-intensive crops (e.g., vegetables, fruits, hops, or specialty teas) where premium quality and labor savings are paramount. Future work should validate this soil-specific approach across various crops, climates, and seasons to develop frameworks that simultaneously enhance sustainability and profitability.

## 5 Conclusions

One-time basal application of specialized fertilizer demonstrates significant potential for enhancing nutrient use efficiency and improving tobacco leaf quality, particularly in middle leaves. Crucially, the economic benefits were profoundly soil-specific. The most significant improvements occurred in sandy soils, driven exclusively by quality-driven value increases rather than yield enhancement. To mitigate the high risk of potassium (K) leaching in sandy soils, we recommend optimizing the fertilizer formulation by increasing the proportion of slow-release K sources or extending the release period to better match the crop’s demand. These findings dictate a soil-specific implementation strategy: specialized fertilizers with enhanced slow-release K properties should be prioritized in sandy soils to maximize economic return, while their value in loamy soils lies primarily in their labor-saving capability while maintaining yield and efficiency. To fully validate and scale this sustainable practice, future work must focus on multi-year trials across diverse agroecological regions and include comprehensive environmental impact assessments.

## Data Availability

The raw data supporting the conclusions of this article will be made available by the authors, without undue reservation.

## References

[B1] Ahmad S. Gao S. Li Q. Nadeem M. Y. Tao W. K. Yang F.. (2023). Controlled-release potassium blended fertilizer mitigates greenhouse gas emissions in paddy fields. Nutr. Cycl. Agroecosyst 127, 1–15. doi: 10.1007/s10705-023-10309-6

[B2] Ahmad S. Nadeem M. Y. Gao S. Li Q. X. Ding Y. F. Liu Z. H.. (2025). Subsurface placement of controlled-release blended fertilizers mitigates ammonia volatilization by promoting nitrogen transformation in rice fields. Agr. Ecosyst. Environ. 386, 109624. doi: 10.1016/j.agee.2025.109624

[B3] Akcura M. Turan V. Kokten K. Kaplan M. (2019). Fatty acid and some micro element compositions of cluster bean (Cyamopsis tetragonoloba) genotype seeds growing under Mediterranean climate. Ind. Crop Prod. 128, 140–146. doi: 10.1016/j.indcrop.2018.10.062

[B4] Bao S. D. (2000). Soil and Agro-Chemistry Analysis. 3rd ed (Beijing: China Agric. Press).

[B5] Bilen S. Bilen M. Turan V. (2019). Relationships between cement dust emissions and soil properties. Pol. J. Environ. Stud. 28, 3089–3098. doi: 10.15244/pjoes/92521

[B6] Cakir R. Cebi U. (2010). The effect of irrigation scheduling and water stress on the maturity and chemical composition of Virginia tobacco leaf. Field Crops Res. 119, 269–276. doi: 10.1016/j.fcr.2010.07.017

[B7] Chamoli A. Bhambri A. Karn S. K. Raj V. (2024). Ammonia, nitrite transformations and their fixation by different biological and chemical agents. Chem. Ecol. 40, 166–199. doi: 10.1080/02757540.2023.2300780

[B8] Cordeiro C. F. S. Rodrigues D. R. Rorato A. F. S. (2022). Cover crops and controlled-release urea decrease need for mineral nitrogen fertilizer for cotton in sandy soil. Field Crops Res. 276, 108387. doi: 10.1016/j.fcr.2021.108387

[B9] Cui P. Chen Z. Ning Q. Wei H. Y. Zhang H. P. Lu H.. (2022). One-time nitrogen fertilizer application using controlled-release urea ensured the yield, nitrogen use efficiencies, and profits of winter wheat. Agronomy 12, 1792. doi: 10.3390/agronomy12081792

[B10] Deng X. H. Zhao S. Y. Zhou Q. M. Zhao S. Y. (2009). Total sugar content of cutter leaf in flue-cured tobacco from Hunan province and its correlation with smoke quality. Acta Tabacaria Sin. 15, 43–47. doi: 10.3969/j.issn.1004-5708.2009.05.010

[B11] Douglas L. A. Riazi A. Smith C. J. (1980). A semi-micro method for determining total nitrogen in soils and plant material containing nitrite and nitrate. Soil Sci. Soc Am. J. 44, 431–433. doi: 10.2136/sssaj1980.03615995004400020047x

[B12] Fan T. F. He M. J. Li C. J. Shi D. X. Yang C. Chen Y. Y.. (2018). Physiological dissection revealed that both uptake and assimilation are the major components regulating different growth responses of two tobacco cultivars to nitrogen nutrition. Plant Biol. 20, 39–49. doi: 10.1111/plb.12642, PMID: 28985445

[B13] Feng X. J. Zhan X. M. Han X. R. Chen K. Peng J. Wang X. X.. (2021). Slow-release nitrogen fertilizer suitable for one-time fertilization of spring maize in Northeast China. Plant Soil Environ. 67, 164–172. doi: 10.17221/162/2020-PSE

[B14] Gil-Ortiz R. Naranjo M.Á. Ruiz-Navarro A. Atares S. García C. Zotarelli L.. (2020). Enhanced agronomic efficiency using a new controlled-released, polymeric-coated nitrogen fertilizer in rice. Plants 9, 1183. doi: 10.3390/plants9091183, PMID: 32932873 PMC7569961

[B15] Grant C. A. Wu R. Selles F. Clayton G. W. Bittman S.. (2012). Crop yield and nitrogen concentration with controlled release urea and split applications of nitrogen as compared to non-coated urea applied at seeding. Field Crop Res. 127, 170–180. doi: 10.1016/j.fcr.2011.11.002

[B16] Gu S. Y. Yu X. W. Jiao Y. S. Zhu Y. W. Zhang Y. H. Zhang H. H.. (2018). Effects of different forms of potassium on plant growth and photosystem II function in leaves of tobacco. Int. J. Agric. Biol. 20, 93–99. doi: 10.17957/IJAB/15.0440

[B17] Guo J. M. Wang Y. H. Blaylock A. D. Chen X. P. (2017). Mixture of controlled release and normal urea to optimize nitrogen management for high-yielding (>15 Mg ha^-1^) maize. Field Crops Res. 204, 23–30. doi: 10.1016/j.fcr.2016.12.021

[B18] He C. P. Liu F. Shi X. Zhao J. J. (2013). Survey and analysis of the current status of tobacco fertilization in Wushan County, Chongqing. Chin. Agric. Sci. Bull. 29, 179–184. doi: 10.3969/j.issn.1000-6850.2013.07.034

[B19] Hei Z. W. Peng Y. T. Hao S. L. Li Y. M. Yang X. Zhu T. B.. (2023). Full substitution of chemical fertilizer by organic manure decreases soil N_2_O emissions driven by ammonia oxidizers and gross nitrogen transformations. Glob. Change Biol. 29, 7117–7130. doi: 10.1111/gcb.16957, PMID: 37800353

[B20] Hei Z. W. Xiang H. M. Zhang J. E. Liang K. M. Zhong J. W. Li M. J.. (2022). Mix-cropping of rice and water mimosa (*Neptunia oleracea Lour.*) increases rice photosynthetic efficiency, yield, grain quality and soil available nutrients. J. Sci. Food Agric. 102, 3972–3982. doi: 10.1002/jsfa.11744, PMID: 34952981

[B21] Hu W. Tian S. B. Di Q. Liu J. Z. Zhang S. X. (2018). Nitrogen mineralization simulation dynamic in tobacco soil. J. Soil Sci. Plant Nutr. 18, 448–465. doi: 10.4067/S0718-95162018005001401

[B22] Hu W. Wei J. Y. Di Q. Tao T. Zhang J. Liu. J.. (2021). Flue-cured tobacco (*Nicotiana tabacum L.*) leaf quality can be improved by grafting with potassium-efficient rootstock. Field Crops Res. 274, 108305. doi: 10.1016/j.fcr.2021.108305

[B23] Hu K. B. Zhao P. Wu K. X. Yang H. L. Yang Q. X. Fan M. P.. (2023). Reduced and deep application of controlled-release urea maintained yield and improved nitrogen-use efficiency. Field Crops Res. 295, 108876. doi: 10.1016/j.fcr.2023.108876

[B24] Huang Z. R. Wu Q. H. Chen Z. L. Wu G. F. Li J. Q. Zhou W. L.. (2023). Varying phosphate fertilizers exerted different effects on inorganic phosphorus transformation, tobacco growth, and phosphorus use efficiency in purple soil. J. Soil Sci. Plant Nutr. 23, 3991–4003. doi: 10.1007/s42729-023-01317-0

[B25] Irfan S. Razali R. KuShaari K. Mansor N. Azeem B. Versypt A. F. (2018). A review of mathematical modeling and simulation of controlled-release fertilizers. J. Control. Release 271, 45–54. doi: 10.1016/j.jconrel.2017.12.017, PMID: 29274697

[B26] Jiang Y. Zhang J. Manuel D. B. Beeck M. O. D. Shahbaz M. Chen Y.. (2022). Rotation cropping and organic fertilizer jointly promote soil health and crop production. J. Enivr. Manage. 315, 115190. doi: 10.1016/j.jenvman.2022.115190, PMID: 35526398

[B27] Kaushik P. (2019). Genetic analysis for fruit phenolics content, flesh color, and browning related traits in eggplant (*Solanum melongena*). Int. J. Mol. Sci. 20 (12), 2990. doi: 10.3390/ijms20122990, PMID: 31248080 PMC6628304

[B28] Kumar A. Bahadur I. Maurya B. R. Raghuwanshi R. Meena V. S. Singh D. K.. (2015). Does a plant growth promoting rhizobacteria enhance agricultural sustainability? J. Pure Appl. Microbiol. 9, 715–724.

[B29] Kurt D. Kinay A. (2021). Effects of irrigation, nitrogen forms and topping on sun cured tobacco. Ind. Crop Prod. 162, 113276. doi: 10.1016/j.indcrop.2021.113276

[B30] Li W. W. Ahmad S. Liu D. Gao S. Wang Y. H. Tao W. K.. (2023). Subsurface banding of blended controlled-release urea can optimize rice yields while minimizing yield-scaled greenhouse gas emissions. Crop J. 11, 914–921. doi: 10.1016/j.cj.2022.10.005

[B31] Li R. C. Gao Y. X. Chen Q. Li Z.L. Gao F. Meng. (2021). Blended controlled-release nitrogen fertilizer with straw returning improved soil nitrogen availability, soil microbial community, and root morphology of wheat. Soil Tillage Res. 212, 105045. doi: 10.1016/j.still.2021.105045

[B32] Liu G. C. Deng L. M. Wu R. J. Guo S. P. Du W. M. Yang M. F.. (2020). Determination of nitrogen and phosphorus fertilisation rates for tobacco based on economic response and nutrient concentrations in local stream water. Agr. Ecosyst. Environ. 304, 107136. doi: 10.1016/j.agee.2020.107136

[B33] Liu A. D. Li Z. H. Zhang D. M. Cui Z. P. Zhan L. J. Xu S. Z.. (2022). One-off basal application of nitrogen fertilizer increases the biological yield but not the economic yield of cotton in moderate fertility soil. Field Crops Res. 288, 108702. doi: 10.1016/j.fcr.2022.108702

[B34] Liu R. M. Wang J. W. Shi J. H. Chen Y. X. Sun C. C. Zhang P. P.. (2014). Runoff characteristics and nutrient loss mechanism from plain farmland under simulated rainfall conditions. Sci. Total Environ. 468-469, 1069–1077. doi: 10.1016/j.scitotenv.2013.09.035, PMID: 24095969

[B35] Liu Z. H. Wu X. B. Tan D. S. Li Y. Jiang L. H. (2018). Application and environmental effects of one-off fertilization technique in major cereal crops in China. Sci. Agric. Sin. 51, 3827–3839. doi: 10.3864/j.issn.0578-1752.2018.20.002

[B36] Liu J. J. Yang T. Z. Zhu B. C. Mei F. Zhang X. Q. (2013). Study on maturity index of flue-cured tobacco leaves based on digital image processing technique. Acta Tabacaria Sin. 19, 61–66. doi: 10.3969/j.issn.1004-5708.2013.03.012

[B37] Ma Q. Qian Y. Yu Q. Cao Y. F. Tao R. G. Zhu M.. (2023). Controlled-release nitrogen fertilizer application mitigated N losses and modified microbial community while improving wheat yield and N use efficiency. Agr. Ecosyst. Environ. 349, 108445. doi: 10.1016/j.agee.2023.108445

[B38] Ma Q. Wang M. Y. Zheng G. L. Yao Y. Tao R. G. Zhu M.. (2021). Twice-split application of controlled-release nitrogen fertilizer met the nitrogen demand of winter wheat. Field Crop Res. 267, 108163. doi: 10.1016/j.fcr.2021.108163

[B39] Meng F. C. Hu K. L. Feng P. Y. Feng G. Z. Gao Q.. (2022). Simulating the effects of different textural soils and N management on maize yield, N fates, and water and N use efficiencies in northeast China. Plants 11, 3338. doi: 10.3390/plants11233338, PMID: 36501377 PMC9741021

[B40] Sakaki T. Fukuhara K. Niino K. Sakuma H. Sugawara S. (1985a). Studies on tobacco aroma.4. Changes in the composition of headspace volatiles of flue-cured tobacco by aging. Agric. Biol. Chem. Tokyo 49, 1785–1791. doi: 10.1271/bbb1961.49.1785

[B41] Sakaki T. Kusama M. Niino K. Sakuma H. Sugawara S.. (1985b). Studies on tobacco aroma.3. Classification of tobaccos with analytical data of nitrogen-containing compounds in their headspace volatiles. Agric. Biol. Chem. Tokyo 49, 1321–1326. doi: 10.1271/bbb1961.49.1321

[B42] Sakaki T. Sakuma H. Sugawara S. (1984). Studies on tobacco aroma.1. Analysis of the headspace volatiles of tobacco using an ether trap. Agric. Biol. Chem. Tokyo 48, 2719–2724. doi: 10.1271/bbb1961.48.2719

[B43] Salehzadeh H. Gholipoor M. Abbasdokht H. Baradaran M.. (2016). Optimizing plant traits to increase yield quality and quantity in tobacco using artificial neural network. Int. J. Plant Prod. 10, 97–108. doi: 10.22069/IJPP.2016.2556

[B44] Schlüter U. Mascher M. Colmsee C. Scholz U. Bräutigam A. Fahnenstich H.. (2012). Maize source leaf adaptation to nitrogen deficiency affects not only nitrogen and carbon metabolism but also control of phosphate homeostasis. Plant Physiol. 160, 1384–1406. doi: 10.1104/pp.112.204420, PMID: 22972706 PMC3490595

[B45] Soares T. D. M. Coelho F. S. Oliveira V. B. Pontes O. Pavinato P. S. (2020). Soil nitrogen dynamics under tobacco with different fertilizer management in southern Brazil. Geoderma Reg. 21, e00282. doi: 10.1016/j.geodrs.2020.e00282

[B46] Subhashini D. V. (2016). Effect of NPK fertilizers and Co-inoculation with phosphatesolubilizing arbuscular mycorrhizal fungus and potassium-mobilizing Bacteria on growth, yield, nutrient acquisition, and quality of tobacco (Nicotiana tabacum L.). Commun. Soil Sci. Plant Anal. 47, 328–337. doi: 10.1080/00103624.2015.1123724

[B47] Sun G. W. Liu Y. Nie W. Du Y. H. Sun J. G. Chen Z. G.. (2024). Multiple omics investigation into the regulatory mechanisms of tobacco growth and quality by transplanting period. Ind. Crop Prod. 217, 118846. doi: 10.1016/j.indcrop.2024.118846

[B48] Thilakarathna S. K. Hernandez-Ramirez G. Puurveen D. Kryzanowski L. Lohstraeter G. Powers L. A.. (2020). Nitrous oxide emissions and nitrogen use efficiency in wheat: N fertilization timing and formulation, soil N, and weather effects. Soil Sci. Soc Am. J. 84, 1910–1927. doi: 10.1002/saj2.20145

[B49] Tso T. C. Mcmurtrey J. E. Sorokin T. (1960). Mineral deficiency and organic constituents in tobacco plants.1. alkaloids, sugars, and organic acids. Plant Physiol. 35, 860–864. doi: 10.1104/pp.35.6.860, PMID: 16655434 PMC406051

[B50] Wang M. Zhang L. He Y. Huang L. K. Liu L. Chen D.. (2022). Soil fungal communities affect the chemical quality of flue-cured tobacco leaves in Bijie, Southwest China. Sci. Rep. 12, 2815. doi: 10.1038/s41598-022-06593-x, PMID: 35181683 PMC8857190

[B51] Whetton R. L. Harty M. A. Holden N. M. (2022). Communicating nitrogen loss mechanisms for improving nitrogen use efficiency management, focused on global wheat. Nitrogen 3, 213–246. doi: 10.3390/nitrogen3020016

[B52] Wu H. L. Wang L. M. Liu X. P Li Q. Lu C. G. Dong W. X.. (2023). Layered-strip fertilization improves nitrogen use efficiency by enhancing absorption and suppressing loss of urea nitrogen. Agron. J. 13, 2428. doi: 10.3390/agronomy13092428

[B53] Wu P. Wu Q. Huang H. Xie L. An H. Y. Zhao X. T.. (2024). Global meta-analysis and three-year field experiment shows that deep placement of fertilizer can enhance crop productivity and decrease gaseous nitrogen losses. Field Crops Res. 307, 109263. doi: 10.1016/j.fcr.2024.109263

[B54] Yan K. Y. (2002). Tobacco Chemistry (Zhengzhou: Zhengzhou University Press).

[B55] Yang X. Y. Geng J. B. Li C. L. Zhang M. Tian X. F. (2016). Cumulative release characteristics of controlled-release nitrogen and potassium fertilizers and their effects on soil fertility, and cotton growth. Sci. Rep. 6, 39030. doi: 10.1038/srep39030, PMID: 27966638 PMC5155277

[B56] Yaseen M. Ahmad A. Naveed M. Ali M. A. Shah S. S. H. Hasnain M.. (2021). Subsurface-applied coated nitrogen fertilizer enhanced wheat production by improving nutrient-use efficiency with less ammonia volatilization. Agrono. J. 11, 2396. doi: 10.3390/agronomy11122396

[B57] Zhang Z. Tian H. Li J. S. Wang D. Wu X. W. (2024). Polyaspartic acid increases potassium content and reduces the ratio of total sugar to nicotine in tobacco leaves. Heliyon 10, e26383. doi: 10.1016/j.heliyon.2024.e26383, PMID: 38444949 PMC10912042

[B58] Zhang L. Zhang X. T. Ji H. W. Wang W. W. Liu J. Wang F.. (2018). Metabolic profiling of tobacco leaves at different growth stages or different stalk positions by gas chromatography–mass spectrometry. Ind. Crop Prod. 116, 46–55. doi: 10.1016/j.indcrop.2018.02.041

[B59] Zhao C. Huang H. Qian Z. H. Jiang H. X. Liu G. M. Xu K.. (2021). Effect of side deep placement of nitrogen on yield and nitrogen use efficiency of single season late japonica rice. J. Integr. Agr. 20, 2–17. doi: 10.1016/S2095-3119(20)63362-7

[B60] Zheng M. Y. Zhu P. Zheng J. Y. Xue L. Zhu Q. F. Cai X. J.. (2021). Effects of soil texture and nitrogen fertilisation on soil bacterial community structure and nitrogen uptake in flue-cured tobacco. Sci. Rep. 11, 22643. doi: 10.1038/s41598-021-01957-1, PMID: 34811391 PMC8608801

[B61] Zhong X. M. Zhou X. Fei J. Y. Wu Y. F. Cao R. L. Huang Y.. (2022). Gained net ecosystem economic benefit in machine - transplanted double-cropped rice strategies. Nutt. Cycling Agroecosyst. 124, 1–15. doi: 10.1007/s10705-022-10218-0

[B62] Zhu X. Y. Chen Y. G. Chen H. Li X. Peng Y. Z. Wang S. Y.. (2013). Minimizing nitrous oxide in biological nutrient removal from municipal wastewater by controlling copper ion concentrations. Appl. Microbiol. Biotechnol. 97, 1325–1334. doi: 10.1007/s00253-012-3988-1, PMID: 22419216

[B63] Zou C. Pearce R. C. Grove J. H. Coyne M. S. (2015). Conservation practices in tobacco production increase large aggregates an associated carbon and nitrogen. Soil Sci. Soc Am. J. 79, 1760. doi: 10.2136/sssaj2015.06.0235

