# Supplementary material for: One-time fertilization in flue-cured tobacco: nutrient dynamics, chemical composition and economic performance across different soil textures
Source: Front Plant Sci. 2025 Oct 6;16:1649093. doi: 10.3389/fpls.2025.1649093 (PMC12536033; doi:10.3389/fpls.2025.1649093)
Supplement: Supplementary file 1 [file DataSheet1.docx]

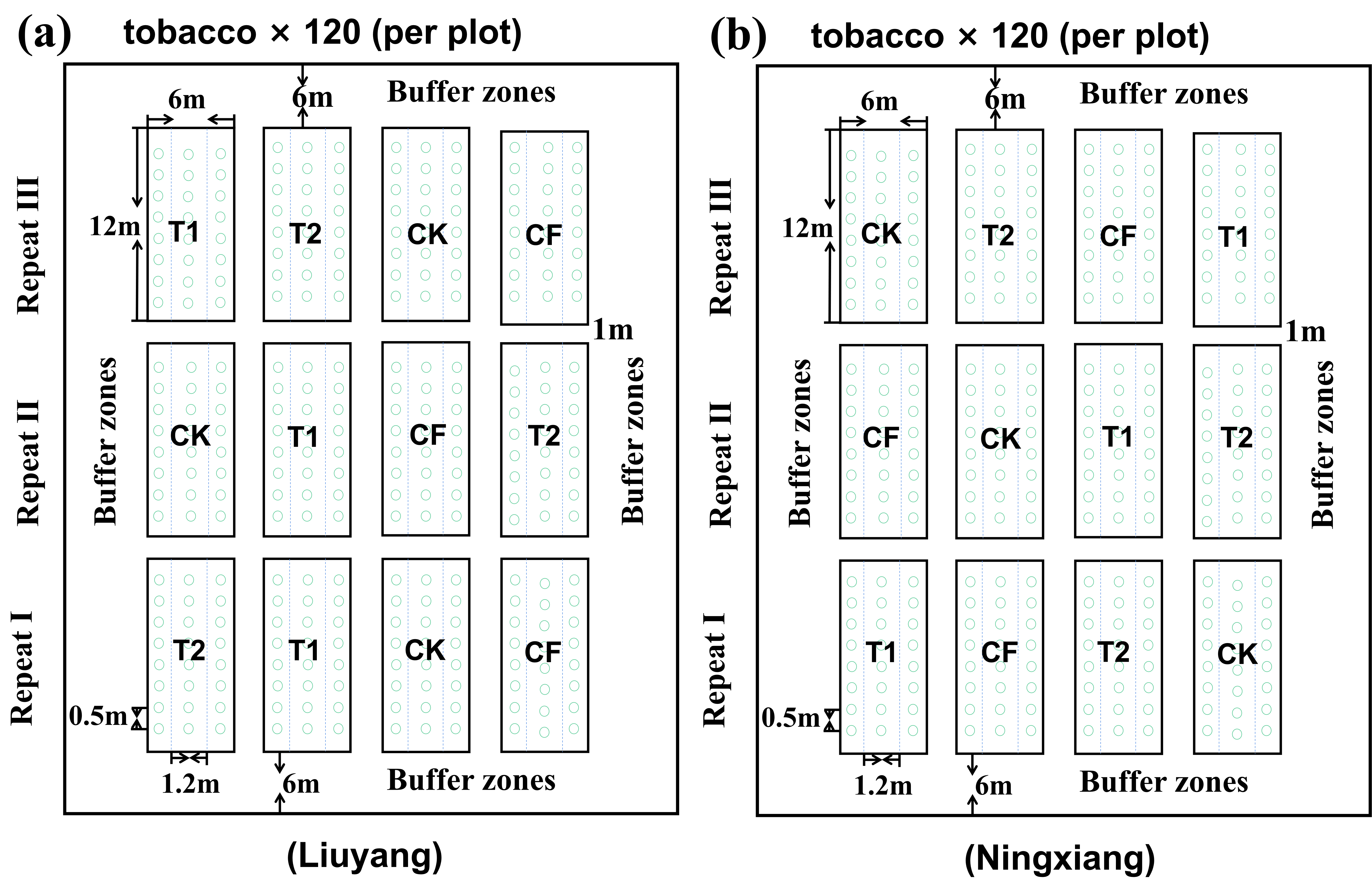


**Figure S1.** Randomized block distribution of experimental plots in Liuyang (a) and Ningxiang (b) areas, plot size, number of tobacco planted in the plots, and spacing between plants and rows

**Text S1.** **Details of fertilizers for testing**

The specialized fertilizer applied in the T1 treatment in the manuscript was a mixture of fast-acting NPK compound fertilizer (10.69-13.12-2.8), slow-release NPK compound fertilizer (10-2.9-21.6), and slow-release potash fertilizer (K_2_O 48%) in the ratio of 2:0.92:1, which was finally formulated into specialized fertilizer Ⅰ (7.8-7.38-18.74).

The specialized fertilizer used in the T2 treatment (excluding the nutrients from the seedling lifting fertilizer) was a mixture of fast-acting NPK composite fertilizer (7.78-10.63-6.88), slow-release NPK composite fertilizer (10-2.9-21.6), and slow-release potash fertilizer (K_2_O 48%) in the ratio 3:1:1, and finally formulated into specialized Fertilizer Ⅱ (7.8-7.38-18.74).

The proportion of slow-release fertilizers in tobacco special fertilizers is 30%. These slow-release fertilizers are polyurethane-coated. From the results, it can be seen that the slow-release nitrogen exhibited an initial faster release phase, with a cumulative release of 42.86% within the first 30 days. This complies with the Chinese national standard for slow-release fertilizers (GB/T 23348-2009), which mandates a 28-day release rate of ≤ 80%. The cumulative release reached 88.49% by day 60, indicating a primary release duration of approximately 60 days in the field soil (Figure S2).

The tobacco-specific fertilizer used in this study was provided by the Institute of Agricultural Resources and Regional Planning, Chinese Academy of Agricultural Sciences; while the fertilizer for the conventional fertilization treatment was provided by the Changsha Tobacco Company.


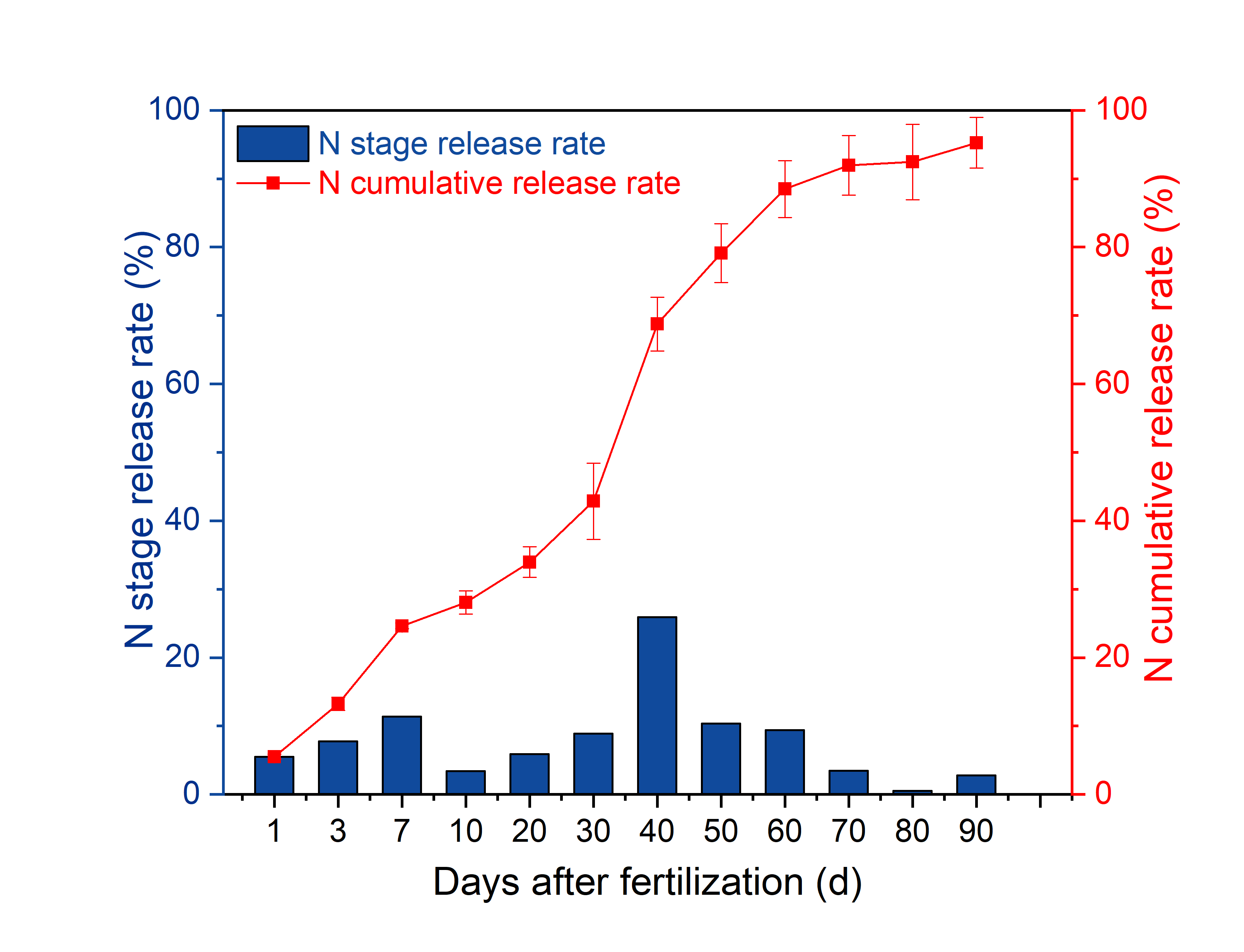


**Figure S2.** Nitrogen accumulative release rate of specialized fertilizer in soil.

**Table S1.** Unit price for different grades of tobacco

|  | tobacco grade | Unit price (yuan/kg) |
| --- | --- | --- |
| top-grade tobacco | C1F | 45.60 |
|  | C2F | 41.40 |
|  | C3F | 36.80 |
|  | B1F | 39.60 |
|  | B2F | 32.80 |
|  | X1F | 32.00 |
| medium-grade tobacco | X2F | 24.00 |
|  | C4F | 26.80 |
|  | X3F | 11.40 |
|  | B3F | 24.20 |
|  | B4F | 11.20 |

**Table S2.** Effect of different fertilizer application methods on total soil nutrients at maturity stage

|  | Treatment | TN（g kg^-1^） | TP（g kg^-1^） | TK（g kg^-1^） | SOM（g kg^-1^） | pH |
| --- | --- | --- | --- | --- | --- | --- |
| Loamy | CK | 1.03±0.09 b | 0.48±0.13 c | 11.55±0.22 d | 17.17±0.78 b | 5.81±0.08 a |
|  | CF | 1.06±0.06 b | 0.69±0.05 bc | 12.37±0.50 c | 17.93±0.32 ab | 5.75±0.11 a |
|  | T1 | 1.21±0.11 a | 0.92±0.22 a | 14.26±0.23 b | 18.52±0.76 a | 5.79±0.04 a |
|  | T2 | 1.19±0.12 a | 0.79±0.12 ab | 15.10±0.32 a | 18.91±1.59 a | 5.75±0.12 a |
| Sandy | CK | 1.06±0.16 b | 0.55±0.05 a | 13.80±1.14 a | 17.76±0.09 a | 5.78±0.13 a |
|  | CF | 1.23±0.17 ab | 0.58±0.17 a | 13.37±1.71 a | 17.30±0.92 a | 5.69±0.14 a |
|  | T1 | 1.27±0.11 a | 0.67±0.01 a | 14.46±1.09 a | 17.48±1.19 a | 5.61±0.14 a |
|  | T2 | 1.24±0.11 a | 0.59±0.07 ab | 14.57±1.17 a | 16.84±0.48 a | 5.59±0.21 a |
